# Quantitative HBV Core Antibodies as a Prognostic Marker for HBeAg Seroclearance: A Systematic Review with Meta-Analysis

**DOI:** 10.3390/v16071121

**Published:** 2024-07-12

**Authors:** Ivana Lazarevic, Danijela Miljanovic, Ana Banko, Maja Cupic, Andja Cirkovic

**Affiliations:** 1 Institute of Microbiology and Immunology, Faculty of Medicine, University of Belgrade, 11000 Belgrade, Serbia; danijela.karalic@med.bg.ac.rs (D.M.); ana.banko@med.bg.ac.rs (A.B.); maja.cupic@med.bg.ac.rs (M.C.); 2 Institute for Medical Statistics and Informatics, Faculty of Medicine, University of Belgrade, 11000 Belgrade, Serbia; andja.cirkovic@med.bg.ac.rs

**Keywords:** hepatitis B virus (HBV), HBV core antibody, quantitation, qAnti-HBc, HBeAg seroclearance

## Abstract

During chronic hepatitis B virus (HBV) infection, the seroclearance of hepatitis B e antigen (HBeAg) is an important event and a significant surrogate endpoint of all current therapeutic strategies. The prediction of HBeAg seroclearance can help assess the benefits of therapy in patients during or before therapy initiation. The quantitation of HBV core antibodies (qAnti-HBc) is a new non-invasive biomarker for solving multiple diagnostic dilemmas. A systematic review and meta-analysis of studies that measured qAnti-HBc in patients who achieved HBeAg seroclearance were performed through PubMed, Web of Science (WoS) and SCOPUS electronic database searches. Nineteen articles were included in the systematic review, comprising 3434 chronically infected patients (1014 with and 2420 without HBeAg seroclearance). Sixteen publications with data regarding qAnti-HBc levels were included in the meta-analysis. The baseline level of qAnti-HBc antibodies was significantly higher in patients with than without HBeAg seroclearance (SMD = 0.88, 95%CI SMD = 0.56–1.2, *p* < 0.001). The same conclusion was reached for patients originating from Asia (SMD = 0.94, 95%CI SMD = 0.55–1.33) and for the qAnti-HBc antibodies among adult HBV patients with therapy-induced HBeAg seroclearance (SMD = 0.90, 95%CI SMD = 0.54–1.25, *p* < 0.001). The systematic review and meta-analysis provide evidence of the role of qAnti-HBc as a promising biomarker for predicting HBeAg seroclearance in chronically infected patients.

## 1. Introduction

According to the World Health Organization (WHO), the number of lives lost due to viral hepatitis is increasing in the newly started decade, and of these, 83% is caused by hepatitis B virus (HBV) [1]. The current estimation is that, worldwide, 254 million people live with chronic HBV infection, and 1.2 million get infected each year. Significant sources of morbidity and mortality are complications related to chronic infection, primarily liver cirrhosis and hepatocellular carcinoma (HCC).

The natural history of chronic HBV infection comprises five distinct but not necessarily successive phases: (1) hepatitis B e antigen (HBeAg)-positive chronic HBV infection, (2) HBeAg-positive chronic hepatitis B (CHB), (3) HBeAg-negative chronic HBV infection, (4) HBeAg-negative active CHB and (5) the HBsAg-negative phase (occult hepatitis) [2]. The distinction between phases and indications for therapy depends on serum HBV DNA and alanine aminotransferase (ALT) levels, the severity of liver disease and the presence of serological markers—HBeAg and hepatitis B surface antigen (HBsAg). The ideal endpoint of both currently available therapies—nucleos(t)ide analogues (NA or NUC) and pegylated interferon alfa (PEG-IFNα)—is a sustained loss of HBsAg with or even without the subsequent appearance of anti-HBs antibodies. However, this endpoint is only occasionlly achieved, and different virological, serological and biochemical markers are used to evaluate therapy success [2,3,4].

HBeAg loss or seroclearance, which can be followed by seroconversion to anti-HBe antibodies, is an important event in the progression of HBV infection and a significant surrogate endpoint of all therapeutic strategies [3,4]. The HBeAg-positive patients without cirrhosis who can achieve HBeAg seroconversion and have HBV DNA undetectability after consolidation therapy are the only group for whom NA therapy cessation is recommended before HBsAg loss [2,3]. Treatment-induced HBeAg seroclearance and seroconversion to anti-HBe marks the elicitation of partial immune control, often leading to a low replicative phase of chronic HBV infection. The spontaneous loss of HBeAg in patients without indications for antiviral therapy also points to more favorable outcomes [5]. Moreover, the initiation of antiviral therapy can be postponed in patients who are likely to achieve spontaneous HBeAg seroclearance. For all these reasons, a need has developed for new non-invasive biomarkers capable of predicting the possibility of HBeAg clearance and seroconversion.

For predicting HBeAg seroclearance in patients’ blood, along with some traditional HBV markers like HBV DNA and HBsAg levels (qHBsAg), some new biomarkers have been investigated, including serum HBV RNA, hepatitis B core-related antigen (HBcrAg) and quantitative HBeAg (qHBeAg) [6,7]. The determination of the anti-HBc level (qAnti-HBc) was shown to provide further information on the status of immune activation in an infected individual and thus can help in the assessment of the phase of infection, stage of liver disease, and ability to reach therapy endpoints [8,9,10]. In this systematic review and meta-analysis, we evaluate the role of anti-HBc quantitation in predicting HBeAg seroclearance.

## 2. Materials and Methods

### 2.1. Study Design

This study, which was previously registered at PROSPERO with the registration number CRD42024546014, was performed according to the recommended PRISMA protocol for systematic reviews and meta-analysis [11].

### 2.2. Eligibility Criteria

Studies that quantified anti-HBc antibodies in HBV patients who had and had not achieved HBeAg seroclearance during the follow-up period were included in this systematic review. Our systematic review considered studies that fulfilled the following inclusion criteria based on the PECOS acronym: (P) population: all human HBV-infected patients (diagnosed as having chronic HBV infection), (E) exposition: HBeAg seroclearance, (C) control: HBeAg persistence, (O) outcome: qAnti-HBc antibodies level, and (S) study design: controlled trials, prospective or retrospective cohort design, nested case–control in cohort design, case–control design, and cross-sectional design. Studies were excluded if they (1) examined populations other than human (animals, cell lines), (2) did not evaluate HBV patients, (3) did not assess qAnti-HBc antibodies, (4) did not evaluate HBeAg seroclearance, (5) did not include a group for comparison, or (6) were not original articles (narrative reviews, systematic reviews, meta-analysis, case reports, case series, editorials, comments, correspondences, books, short, abstracts, etc.).

Two researchers with expertise in conducting systematic reviews and meta-analyses (AC, IL) developed and ran the search in three electronic databases: PubMed, Web of Science (WoS), and SCOPUS until 22 March 2024. The following keywords were used for the search query in PubMed: (“quantitative HBV core antibodies” OR “qAnti-HBc” OR “quantitative anti-HBc” OR “quantitative HBcAb” OR “qHBcAb” OR “level anti-HBc” OR “level HBcAb” OR “quantitation anti-HBc” OR “quantification anti-HBc” OR “quantitation HBV core antibodies” OR “anti-HBc” OR “HBcAb”) AND (“HBeAg seroconversion” OR “HBeAg loss” OR “HBeAg seroclearance” OR “Hepatitis B e antigen loss” OR “Hepatitis B e antigen seroclearance” OR “treatment outcome” OR “serological response” OR “SR”), in SCOPUS: (TITLE-ABS-KEY (“quantitative HBV core antibodies”) OR TITLE-ABS-KEY (“qAnti-HBc”) OR TITLE-ABS-KEY (“quantitative anti-HBc”) OR TITLE-ABS-KEY (“quantitative HBcAb”) OR TITLE-ABS-KEY (“qHBcAb”) OR TITLE-ABS-KEY (“level anti-HBc”) OR TITLE-ABS-KEY (“level HBcAb”) OR TITLE-ABS-KEY (“quantitation anti-HBc”) OR TITLE-ABS-KEY (“quantification anti-HBc”) OR TITLE-ABS-KEY (“quantitation HBV core antibodies”) OR TITLE-ABS-KEY (“anti-HBc”) OR TITLE-ABS-KEY (“HBcAb”)) AND (TITLE-ABS-KEY (“HBeAg seroconversion”) OR TITLE-ABS-KEY (“HBeAg loss”) OR TITLE-ABS-KEY (“HBeAg seroclearance”) OR TITLE-ABS-KEY (“Hepatitis B e antigen loss”) OR TITLE-ABS-KEY (“Hepatitis B e antigen seroclearance”) OR TITLE-ABS-KEY (“treatment outcome”) OR TITLE-ABS-KEY (“serological response”) OR TITLE-ABS-KEY (“SR”)), and in WoS: (ALL = (“quantitative HBV core antibodies”) OR ALL = (“qAnti-HBc”) OR ALL = (“quantitative anti-HBc”) OR ALL = (“quantitative HBcAb”) OR ALL = (“qHBcAb”) OR ALL = (“level anti-HBc”) OR ALL = (“level HBcAb”) OR ALL = (“quantitation anti-HBc”) OR ALL = (“quantification anti-HBc”) OR ALL = (“quantitation HBV core antibodies”) OR ALL = (“anti-HBc”) OR ALL = (“HBcAb”) AND (ALL = (“HBeAg seroconversion”) OR ALL = (“HBeAg loss”) OR ALL = (“HBeAg seroclearance”) OR ALL = (“Hepatitis B e antigen loss”) OR ALL = (“Hepatitis B e antigen seroclearance”) OR ALL = (“treatment outcome”) OR ALL = (“serological response”) OR ALL = (“SR”). Publications in English were only considered. In addition, reference lists of the articles identified through electronic retrieval and relevant reviews and editorials were manually searched to check for more potentially relevant articles.

### 2.3. Article Screening and Selection

Publications were screened for inclusion in the systematic review independently by two reviewers (DM, AB), first through the title and abstract and afterwards through full-text reading. All disagreements were resolved by discussion at each stage with the inclusion of a third reviewer (IL). Studies were included in the full-text screening step if either reviewer identified the study as potentially eligible or if the abstract and title did not have sufficient information for exclusion.

### 2.4. Data Abstraction and Quality Assessment

Two reviewers (IL, AC) independently abstracted the following data: author(s), year of publication, country of research, study design, population characteristics (size, age, gender, origin, HIV and HDV coinfection, complications like cirrhosis or hepatocellular carcinoma (HCC), HBV genotype, baseline values (HBV DNA, HBsAg level, HBV RNA, HBcrAg, HBeAg level, aspartate aminotransferase—AST, ALT), number of patients with and without HBeAg seroclearance, type of seroclearance, duration of follow-up, method and manufacturer of qAnti-HBc antibodies quantification, qAnti-HBc antibody class, baseline value of qAnti-HBc antibodies, antiviral therapy and its duration. Independent reviewers used previously designed protocols to select and abstract data. Authors of the relevant articles were contacted to obtain unavailable manuscripts and/or missing data. Each reviewer independently evaluated the quality of chosen manuscripts using an adapted version of the Newcastle–Ottawa tool (NOS) for observational studies [12] and the Jadad scale for controlled trials [13]. The study quality, according to NOS, was good (3 or 4 stars in selection and 1 or 2 stars in comparability and 2 or 3 stars in outcome/exposure domain or ≥7 stars in total), fair (2 stars in selection and 1 or 2 stars in comparability and 2 or 3 stars in outcome/exposure domain or 5–6 stars in total), or poor (0 or 1 star in selection or 0 stars in comparability or 0 or 1 star in outcome/exposure or ≤4 stars in total). Jadad could have 5 points in total, and the categorization was as follows: 0–2: low and 3–5: high quality.

### 2.5. Statistical Analysis

The primary goal of this study was to evaluate the level of qAnti-HBc antibodies in chronically infected patients with and without HBeAg seroclearance. It was achieved by the standardized mean difference (SMD) as the measure of effect size, with the fixed or random effect method, depending on the heterogeneity of the included studies. Heterogeneity was evaluated by the chi-square Q test and I^2^ statistic that presents the inconsistency between the study results and quantifies the proportion of observed dispersion that is real, i.e., due to between-study differences and not a random error. Heterogeneity was present if the chi-square Q test was significant with *p* ≤ 0.05. The categorization of heterogeneity was based on the Cochrane Handbook [14] and stated that I^2^ < 30%, 30% to 60% and >60% corresponded to low, moderate and high heterogeneity, respectively. The random effect model was applied in the case of high heterogeneity. The median was used to approximate the arithmetic mean, and IQR/1.35 was used to approximate the standard deviation. If a standard error was used in the original article, the standard deviation was calculated as sd = se*√n, and if the range was presented, the standard deviation was estimated as (max − min)/4. Forest plots were constructed for each outcome reporting SMD (box), 95% confidence interval (lines) of the SMD, and weight (size of box) for each trial. A diamond represented the overall effect size with its 95% confidence interval. Funnel plots assessed publication bias for each outcome (Supplementary Materials, Figures S1–S8). A *p*-value ≤ 0.05 was considered to be statistically significant. Analyses were performed using Review Manager 5.4 [15].

## 3. Results

### 3.1. Study Characteristics

**Figure 1:**
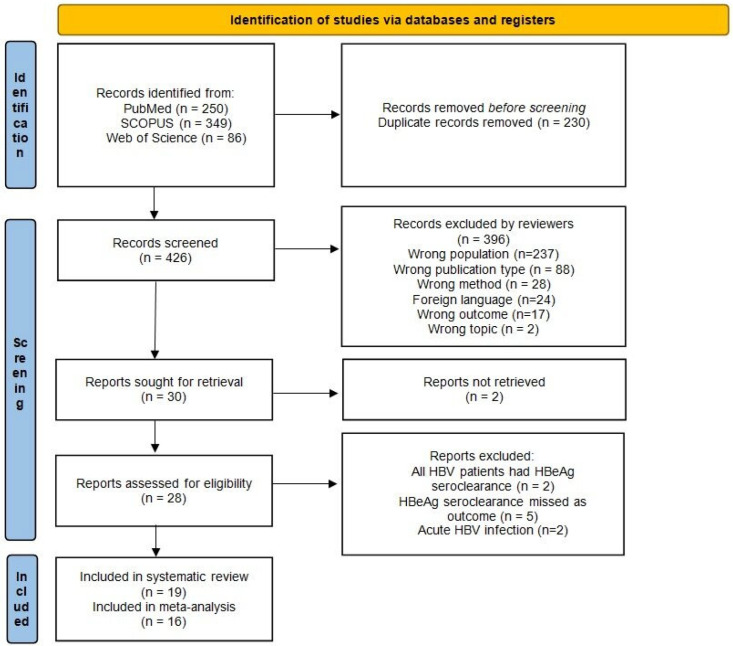
The flow chart represents the search strategy and study selection for the systematic review.

A total of 685 potentially eligible articles were found. Title and abstracts were evaluated for 426 articles after duplicates (260) were removed. A total of 30 were retrieved, 29 were considered in full text after 230 articles were excluded according to the previously defined exclusion criteria, and two were unavailable. Finally, 19 articles were selected for inclusion in the systematic review and 16 for meta-analysis. A flow diagram illustrating the selection process is presented in Figure 1.

Characteristics of the 19 included publications within the systematic review are presented in Table 1. They were published between 2015 and 2022, with a total of 3434 HBV patients (1014 with and 2420 without HBeAg seroclearance). The minimum sample size was nine for HBV patients with and seven for HBV patients without HBeAg seroclearance. There were three clinical trials, six retrospective cohorts, and four prospective cohorts. In six publications, the study design was not reported. Most studies were from China (16). One study each was conducted in Taiwan, France, and the Netherlands. Adults were the population of interest in all included publications, while children were examined in only one study. There were 2618 male and 817 female HBV patients. The examined population originated mainly from Asia (16/19). The population was multicenter and unclear in two studies. The most common HBV genotypes were C (in 1258 patients) and B (in 971 patients). Other genotypes (A, D, E, G, and mixed B + C) were detected in 342 patients. The HBV genotype was not reported in 7/19 publications. Possible HIV coinfection was researched in 10/19, while HBV patients with HIV coinfection were examined in just 1 publication, and in the other 9 publications, HBV patients were HIV-negative. Possible HDV coinfection was investigated in 13/19 publications; in all of them, HBV patients were HDV-negative. HIV and HDV coinfections were not reported in 9/19 and 6/19 publications, respectively. HBV patients with cirrhosis were evaluated in 1 publication; cirrhosis was absent in 8/19, and this complication of chronic infection was not reported in 10/19 publications. All included studies evaluated qAnti-HBc antibodies measured as the total Anti-HBc level. Therapy-induced HBeAg seroclearance was evaluated in 18/19 included studies, which comprised 910 HBV patients, while spontaneous HBeAg seroclearance was examined in only 1 study comprising 182 HBV patients. Commonly applied therapy was entecavir (ETV) (9/18), then pegylated interferon (PEG IFN) (7/18), tenofovir (TNF) (4/18), interferon alfa (IFNα) (1/18), and lamivudine (LAM) + adefovir (ADF) (3/18). NUC, ADF, and telbivudine (LdT) as single therapies, and combinations of LAM + TNF and IFN + LAM were applied in one study each. The duration of follow-up until HBeAg seroclearance differed from a minimum of 18 weeks to a maximum of 24 years.

Additional relevant results were collected from the included studies and are listed in the Supplementary Materials, Table S1. They include values of AST, ALT, HBV DNA, HBsAg, HBV RNA, HBcrAg and HBeAg levels in the overall observed population, as well as in the HBeAg seroclearance group and group without HBeAg seroclearance. The group with seroclearance had higher values of ALT (10/12 studies), AST (3/4 studies) and HBV RNA (1/1 study), while the values of HBV DNA (8/12 studies), HBsAg (11/11 studies), HBeAg (7/8 studies) and HBcrAg (2/2 studies) were lower in this group. Most included studies reported the continuous decline in qAnti-HBc levels during therapy (15/19 studies). A decline was observed in both groups, with qAnti-HBc levels remaining higher in the seroclearance group. HBsAg clearance was achieved in 32 patients of the HBeAg seroclearance group.

#### Quality Assessment

We performed a quality assessment of 17 included studies in the quantitative analysis using the NOS system for cohorts and the Jadad scale for clinical trials. The details of the star system are shown in Table 2. Almost all studies were of good quality (15/17), while 1 was of fair quality and 1 was of poor quality.

### 3.2. Meta-Analysis Results

Sixteen publications with available data regarding the level of qAnti-HBc antibodies were included in the meta-analysis of the difference in the level of anti-HBc antibodies among HBV patients with and without spontaneous or therapy-induced HBeAg seroclearance.

**Figure 2:**
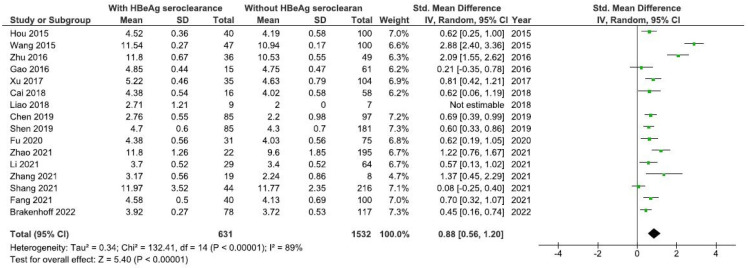
Meta-analysis of the differences in the baseline level of the quantitation of HBV core antibodies (qAnti-HBc) antibodies between HBV patients with and without hepatitis B e antigen (HBeAg) seroclearance [8,17,18,19,20,21,22,23,24,25,27,29,30,31,34].

**Figure 3:**
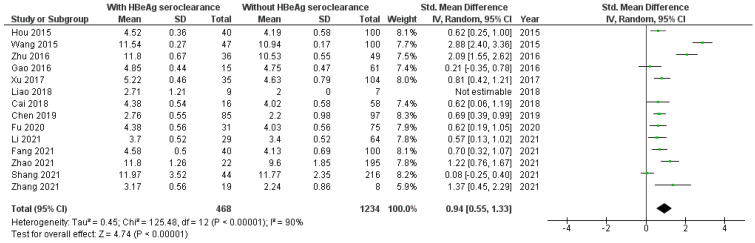
Meta-analysis of the differences in the level of qAnti-HBc antibodies between HBV patients originating from Asia with and without HBeAg seroclearance [8,17,18,19,20,21,22,23,24,27,29,30,31].

**Figure 4:**
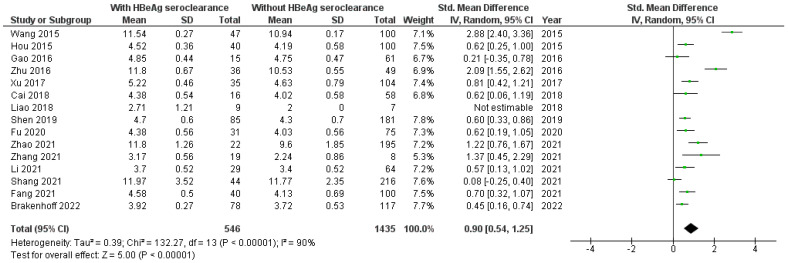
Meta-analysis of the differences in the level of total qAnti-HBc antibodies between adult HBV patients with and without therapy-induced HBeAg seroclearance [8,17,18,19,20,21,22,23,25,27,29,30,31,34].

**Figure 5:**
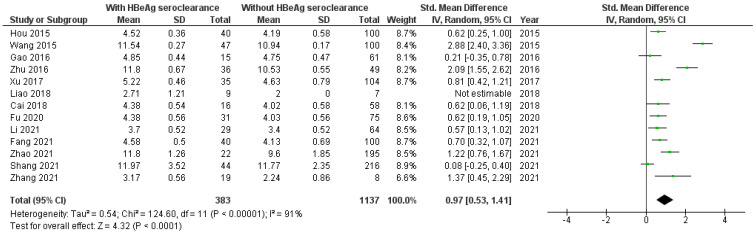
Meta-analysis of the differences in the level of qAnti-HBc antibodies between adult HBV patients originating from Asia with and without therapy-induced HBeAg seroclearance [8,17,18,19,20,21,22,23,27,29,30,31].

According to 16 included studies [8,17,18,19,20,21,22,23,24,25,27,29,30,31,34] the baseline level of qAnti-HBc antibodies was significantly higher in HBV patients with than without HBeAg seroclearanc (SMD = 0.88, 95%CI SMD = 0.56–1.20, *p* < 0.001) (Figure 2). It was significantly higher in HBV patients originating from Asia with than without HBeAg seroclearance [8,17,18,19,20,21,22,23,24,27,29,30,31] (SMD = 0.94, 95%CI SMD = 0.55–1.33, *p* < 0.001) (Figure 3). Also, the level of qAnti-HBc antibodies was significantly higher in (1) adult HBV patients with therapy-induced HBeAg seroclearance [8,17,18,19,20,21,22,23,25,27,29,30,31,34] (SMD = 0.90, 95%CI SMD = 0.54–1.25, *p* < 0.001) (Figure 4) and (2) in adult HBV patients originating from Asia with therapy-induced HBeAg seroclearance [8,17,18,19,20,21,22,23,27,29,30,31] (SMD = 0.97, 95%CI SMD = 0.53–1.41, *p* < 0.001) (Figure 5).

Sensitivity analysis for all previous comparisons was performed by excluding studies that had reported qAnti-HBc antibodies level as S/Co rate. The conclusions were the same, and they were given for (1) the differences in the baseline level of qAnti-HBc antibodies between HBV patients with and without HBeAg seroclearance (SMD = 0.65, 95%CI SMD = 0.50–0.73, *p* < 0.001) (Supplementary Materials, Figure S9), (2) the differences in the level of qAnti-HBc antibodies between HBV patients originating from Asia with and without HBeAg seroclearance (SMD = 0.66, 95%CI SMD = 0.52–0.80, *p* < 0.001) (Supplementary Materials, Figure S10), (3) the differences in the level of total qAnti-HBc antibodies between adult HBV patients with and without therapy-induced HBeAg seroclearance (SMD = 0.60, 95%CI SMD = 0.48–0.73, *p* < 0.001) (Supplementary Materials, Figure S11), and (4) the differences in the level of qAnti-HBc antibodies between adult HBV patients originating from Asia with and without therapy-induced HBeAg seroclearance (SMD = 0.65, 95%CI SMD = 0.49–0.81, *p* < 0.001) (Supplementary Materials, Figure S12).

## 4. Discussion

HBeAg is a non-structural protein generated by the maturation of the pre-core/core precursor molecule in the endoplasmic reticulum and secreted as a soluble antigen in the circulation. Although not required for viral replication or infection, it plays a pivotal role in the viral–host interplay and the establishment of chronic HBV infection [35]. Since its discovery, it has been regarded as an additional marker of active viral replication. Today, it is crucial in identifying the phases of chronic infection, and it represents one of the endpoints of both available therapeutical options [2,3,4]. The disappearance of HBeAg can, in some cases, result from the emergence of an HBV basal core promotor and/or pre-core mutations when it is followed by the establishment of active chronic hepatitis and the progression of liver disease [2,35]. However, HBeAg seroclearance, especially accompanied by the appearance of anti-HBe antibodies (seroconversion), is an indication of favorable outcomes in both treated and untreated patients [2,3,4,5].

HBeAg seroclearance signals a transition from an immunologically active phase to a low replicative phase of chronic infection in both treated and untreated patients. This phase is characterized by lower HBV DNA levels, the normalization of ALT levels, minimal hepatic necroinflammatory activity and the low risk of progression to cirrhosis or HCC. Patients who achieve HBeAg seroconversion are also candidates for HBsAg seroclearance, known as a functional cure [2]. HBeAg seroconversion is the only indication for NA therapy cessation before HBsAg seroclearance in eligible patients. The prediction of HBeAg seroclearance can help assess the risks and benefits in patients during or even before the initiation of therapy [5,36]. The recognized predictors include lower HBV DNA levels, higher ALT levels and older age [5,37]. Among many new HBV biomarkers developed to help settle diagnostic dilemmas, the predictive value of HBcrAg and HBV RNA was also suggested [38]. In addition, more rapid HBeAg loss is associated with lower baseline HBsAg levels and an early decline in this level in patients on therapy [39,40]. Interestingly, it was recently found that levels of small (LHB) and middle (MHB) surface proteins decrease earlier than total HBsAg in patients who will eventually experience a functional cure [41]. The authors noticed that MHB was a more sensitive biomarker than total HBsAg or LHB level for predicting HBeAg seroconversion, showing low levels (<1%) or complete loss in more than 50% of patients who achieved it.

The presence of antibodies to the HBV core antigen is a classical serological marker used to identify individuals who were ever infected with HBV. Anti-HBc can be found in the serum of an infected individual very soon after the detection of HBsAg and can persist continuously for decades. It is a marker present in nearly 100% of chronically infected patients (in all phases of chronic HBV infection) and 80–99% of individuals with occult infection [42,43]. The level of anti-HBc antibodies (qAnti-HBc) can indicate the status of immune activation in an infected individual and can also be a surrogate marker for the intrahepatic HBV core antigen load [44]. Its ability to help assess the phase of infection, the stage of liver inflammation and fibrosis and to predict therapy endpoints has been investigated in recent years.

This meta-analysis, which comprised 16 studies, investigated the difference in the baseline level of anti-HBc antibodies among HBV patients with and without spontaneous or therapy-induced HBeAg seroclearance. The main finding was that the baseline level of qAnti-HBc antibodies was significantly higher in HBV patients with than without HBeAg seroclearance. The applied therapy included both therapy strategies (NA and IFN) as well as their combination. The same conclusion was reached for patients of Asian origin and adult patients with therapy-induced HBeAg seroclearance. The favorable outcome of infection, marked by HBeAg seroclearance, in treated and untreated patients is associated with a higher level of the patient’s adaptive immune status. Higher antibody levels are the result of higher B-cell activation, which is also related to higher levels of cytokines responsible for the activity of CD4+ and CD8+ T-cells [45]. On the other hand, it should be noted that high qAnti-HBc levels correlate positively with the severity of liver inflammation and can obstruct the improvement in inflammation severity in patients receiving therapy [46,47]. This is because HBcAg is released upon the destruction of hepatocytes and is responsible for the stimulation of HBcAg-specific B-cells. Similarly, in patients who have reached the HBsAg-negative stage, a high anti-HBc level is linked with occult infection and the possibility of reactivation [48].

Nineteen studies included in the systematic review reported factors associated with HBeAg seroclearance, and in fifteen, the qAnti-HBc baseline level was named an important factor for predicting HBeAg seroclearance. While in most studies, the level of anti-HBc antibodies was combined with other parameters for a stronger prediction value, in two studies, the qAnti-HBc level was indicated as the only independent predictor [8,22]. The other parameters combined with qAnti-HBc most often included ALT and HBeAg levels, and in some studies, HBV DNA and HBsAg levels, age, gender, viral genotypes B and B + C, treatment time, anti-HBe antibodies, liver stiffness measurement (LSM), triglyceride (TG) and alkaline phosphatase (AP) levels as well as two new biomarkers HBcrAg and HBV RNA. Based on this and similar to other new HBV diagnostic markers, combining qAnti-HBc with other parameters warrants more reliable predictive power [6,7,38].

Most included studies reported the continuous decline in qAnti-HBc levels during therapy. A decline was observed in both groups, with and without seroclearance, with qAnti-HBc levels remaining higher in the seroclearance group. Also, the decline was steeper in patients treated with NA than IFN [16,17,34]. This can be explained by the effect of IFN being the immunomodulating drug and inducing a more robust immune response, including anti-HBc. In accordance with this, one of the studies that measured IgM anti-HBc reported that IFN treatment initially caused an increase in serum anti-HBc IgM, after which these antibodies steadily declined [49]. One of the included studies reported the rebound of qAnti-HBc titer during follow-up, but the rebound was milder in patients with higher baseline levels [17].

The baseline qAnti-HBc level was reported to correlate with ALT, CD4+ T-cell count and older age, while some slight correlation was found with HBsAg levels, HBcrAg, HBV RNA and LSM. Although ALT level is considered a surrogate marker of anti-HBV activity and was shown to be a strong predictor of HBeAg clearance, qAnti-HBc was demonstrated to be superior in this task, probably due to its HBV specificity [17,21].

Among the observed studies, the most often used methods for anti-HBc quantitation are the double-sandwich immunoassay and the chemiluminescent microparticle immunoassay (CMIA). The double-sandwich immunoassay, first designed in 2010, was reported to be superior to others in sensitivity and specificity [50]. It includes calibration against the WHO International Standard, and results can be reported as IU/mL. However, other methods are only semi-quantitative and demand calibration against the standard to express results in international units. Thus, the anti-HBc values were expressed as sample/cut-off (S/Co) values or as units according to the previous Paul Erich Institute standard (PEIU/mL). This seriously hindered comparing results from different studies and prevented a definite conclusion on the universal qAnti-HBc cut-off value for HBeAg seroclearance.

Another limitation of this systematic review was that most studies included populations of Asian origin with the domination of B, C and mixed B and C genotype infections. Although anti-HBc antibodies are genotype-independent markers, insight into populations from different continents and infected with various genotypes would strengthen conclusions.

## 5. Conclusions

In summary, the systematic review and meta-analysis provide valuable evidence of the role of HBV core antibody quantitation as a promising new non-invasive biomarker for predicting HBeAg seroclearance in chronically infected patients. Given the lack of uniformity in the published studies regarding applied methods, quantitation unit cut-off values and the predominance of the Asian population, future research is warranted to assess the validity and reliability of anti-HBc quantitation as a prognostic marker.

## Figures and Tables

**Table 1:** The systematic review—study characteristics.

Study Characteristics	HBV Patients						qAnti-HBc			HBeAg Seroclearance				
Author, Year, Country Study Design	*n*	Age * (Years)	Gender M/F	Origin	HIV Coinfection HDV Coinfection Cirrhosis/HCC	HBV Genotype	Class Unit of qAnti-HBc	qAnti-HBc Method (Manufacturer)	Cut-Off Value of qAnti-HBc for HBeAg Seroclearance	*n* with HBeAg Seroclearance Value of qAnti-HBc **	*n* without HBeAg Seroclearance Value of qAnti-HBc **	Type of HBeAg Seroclearance	Therapy Duration	Duration of Follow-Up
Fan, 2015 [16], China Retrospective cohort	231 PEG IFN cohort 560 NUC cohort	PEG IFN cohort 29.1 ± 6.8 NUC cohort 30.1 ± 8.9	PEG IFN cohort 184/47 NUC cohort 455/105	Asia (China)	NR NR NR	PEG IFN cohort: 81 B, 148 C, 2 others NUC cohort: 217 B, 340 C, 3 others	total anti-HBc log IU/mL	double-sandwich immunoassay (Wantai, Beijing, China)	>4.4 log IU/mL baseline qAnti-HBc for prediction of HBeAg seroclearance for both cohorts, together with baseline HBV DNA < 9 log copies/mL	99 (PEG IFN cohort), 137 (NUC cohort) higher levels in HBeAg seroclearance cohort than in cohort without HBeAg seroclearance	132 (PEG IFN cohort), 423 (NUC cohort) higher levels in HBeAg seroclearance cohort than in cohort without HBeAg seroclearance	therapy-induced	PEG IFN, NUC 24 weeks (PEG IFN cohort), 104 weeks (NUC cohort)	24 weeks (PEG IFN cohort), 104 weeks (NUC cohort)
Hou, 2015 [17], China Multicenter, randomized, double-blind, controlled phase II clinical trial	140	overall 26.75 ± 6.99 seroclearance 25.32 ± 6.72 without seroclearance 27.32 ± 7.04	overall 103/37 seroclearance 27/13 without seroclearance 76/24	Asia (China)	No No NR	overall 51B, 89C seroclearance 16B, 24C without seroclearance 35B, 65C	total anti-HBc log IU/mL	double-sandwich immunoassay (Wantai, Beijing, China)	>4.47 log IU/mL (30,000 IU/mL)–higher % of HBeAg seroclearance	40 4.52 ± 0.36	100 4.19 ± 0.58	therapy-induced	PEG IFN 48 weeks	72 weeks
Wang, 2015 [18], China Prospective cohort	147	overall 25.02 ± 0.56 seroclearance 26.40 ± 1.19 without seroclearance 24.37 ± 0.59	overall 106/41 seroclearance 33/14 without seroclearance 73/27	Asia (China)	No No NR	overall 85B, 55C, 7 other seroclearance 26B, 21C without seroclearance 59B, 34C, 7 other	total anti-HBc, S/Co	commercially available kit (Abbott, Chicago, IL, USA)	>11.4 S/Co	47 11.54 ± 0.27	100 10.94 ± 0.17	therapy-induced	IFN alfa 1b 52 weeks	52 weeks
Gao, 2016 [19], China Retrospective cohort	76	overall 32.63 ± 9.69 seroclearance 31.87 ± 11.6 without seroclearance 32.82 ± 9.26	overall 56/20 seroclearance 10/5 without seroclearance 46/15	Asia (China)	NR NR NR	overall 53C, 23 other seroclearance 10C, 5 other without seroclearance 43C, 18 other	total anti-HBc, log IU/mL	double-sandwich immunoassay (Wantai, Beijing, China)	NR	15 4.85 ± 0.44	61 4.75 ± 0.47	therapy-induced	LAM + ADF >96 weeks	96 weeks
Zhu, 2016 [20], China Prospective cohort	85	overall 23.85 ± 1.15 seroclearance 23.50 ± 1.92 without seroclearance 24.10 ± 1.46	overall 66/19 seroclearance 27/9 without seroclearance 39/10	Asia (China)	No No NR	overall 50B, 31C, 4 other seroclearance 21B, 13C, 2 other without seroclearance 29B, 18C, 2 other	total anti-HBc S/Co	commercially available enzyme immunoassay (Abbott, Chicago, IL, USA)	baseline > 10.7 S/Co together with HBeAg ≤ 500 S/Co and ALT > 5 × ULN	36 11.80 ± 0.67	49 10.53 ± 0.55	therapy-induced	PEG IFN alfa 2a 52 weeks	76 weeks
Xu, 2017 [21], China Retrospective cohort	139	overall 30.91 ± 8.92 seroclearance 30.23 ± 9.66 without seroclearance 31.44 ± 8.80	overall 110/29 seroclearance 19/16 without seroclearance 91/13	Asia (China)	NR NR NR	overall 81B, 58C seroclearance 16B, 19C without seroclearance 39B, 65C	total anti-HBc, log IU/mL	double-sandwich immunoassay (Wantai, Beijing, China)	4.65 log IU/mL	35 5.22 ± 0.46	104 4.63 ± 0.79	therapy-induced	ETV 240 weeks	240 weeks
Cai, 2018 [22], China Multicenter, randomized, controlled clinical trial	74 Total 32 LAM + ADF group 42 ETV group	LAM + ADF group 32.9 ± 10.6 ETV group 30.1 ± 8.4	LAM + ADF group 25/7 ETV group 31/11	Asia (China)	No No No	NR	total anti-HBc log IU/mL	double-sandwich immunoassay (Wantai, Beijing, China)	>4.375 log IU/mL, for HBeAg seroclearance at week 96 (40% of those with >4.375 log IU/mL had seroclearance, 12.2% with lower level had seroclearance)	16 baseline, 4.38 ± 0.54	58 4.02 ± 0.58	therapy-induced	LAM + ADF (32), ETV (42) 96 weeks	96 weeks
Liao, 2018 [23], China NR	16	seroclearance 49 (38–55) without seroclearance 42 (31–43)	seroclearance 9/0 without seroclearance 7/0	Asia (China)	No No NR	NR	total anti-HBc log IU/mL	double-sandwich immunoassay (Wantai, Beijing, China)	NR	9 baseline, 2.71 (2.00, 3.63)	7 2.00 (2.00, 2.00)	therapy-induced	NA (LAM/ADF/ETV) Long term	NR
Chen, 2019 [24], Taiwan NR	182	overall 10.6 (11.9–21.9) seroclearance 10.62 (10.35–15.39) without seroclearance 10.52 (8.98–15.26)	overall 106/76 seroclearance 46/39 without seroclearance 60/37	Asia (Taiwan)	NR NR No	overall 128B, 42C, 12 mixed B + C seroclearance 6B, 12C, 11 mixed B + C without seroclearance 66B, 30C, 11 mixed B + C	total anti-HBc log IU/mL	double-sandwich immunoassay (Wantai, Beijing, China)	>2.7 log IU/mL (500 IU/mL)	85 baseline 2.76 (2.23–2.97)	97 2.20 (1.39–2.71)	spontaneous	No /	9–24 years
Shen, 2019 [25], China Retrospective cohort	526 Total 258 training cohort 268 validation cohort	overall training cohort 40.3 ± 11.2 seroclearance training cohort 39.2 ± 11.5 without seroclearance training cohort 40.9 ± 11.0 overall validation cohort 38.5 ± 10.9 seroclearance validation cohort 36.1 ± 8.8 without seroclearance validation cohort 39.6 ± 11.7	overall training cohort 192/66 seroclearance training cohort 68/25 without seroclearance training cohort 124/41 overall validation cohort 216/52 seroclearance validation cohort 73/14 without seroclearance validation cohort 143/38	not clear training cohort: Asia (China) *n* = 252 validation cohort: Asia (China) *n* = 253	NR NR cirrhosis training cohort: *n* = 53 overall *n* = 22 seroclearance *n* = 31 without seroclearance validation cohort: *n* = 49 overall *n* = 14 seroclearance *n* = 35 without seroclearance	Training cohort: 2A, 111B, 141C, 2D, 1E, 1G Validation cohort: 5A, 116B, 137C, 7D, 2E, 1G	total anti-HBc log IU/mL	double-sandwich immunoassay (Wantai, Beijing, China)	Cut-off only in predictive model including age, anti-HBc and HBsAg	93 (training cohort) 87(validation cohort) 4.5 ± 0.7 (training cohort) 4.7 ± 0.6 (validation cohort)	165 (training cohort) 181 (validation cohort) 4.3 ± 0.6 (training cohort) 4.3 ± 0.7 (validation cohort)	therapy-induced	ETV 4.47 (1–10.58) (training cohort) 4.30 (1–9.76) (validation cohort)	2.75 (0.05–5.00) (training cohort) 2.56 (0.02–5.00) (validation cohort)
Dezanet, 2020 [26], France Prospective cohort	95	40.3 (35.0–46.7)	89/6	not clear (mostly Europe—France)	Yes No NR	55A, 7D, 4E, 12G	total anti-HBc log PEIU/mL	CMIA (Abbott, Chicago, IL, US)	>4.1 log PEIU/mL and HBcrAg < 7.5 U/mL	26 NR	69 NR	therapy-induced	28 TNF, 67 LAM + TNF >6 months	8 years
Fu, 2020 [27], China Prospectve cohort	106	overall 34.4 ± 10.7 years seroclearance 40.7 ± 10.5 without seroclearance 31.8 ± 9.6	seroclearance 24/7 without seroclearance 65/10	Asia (China)	NR No No	NR	total anti-HBc log IU/mL	double-sandwich immunoassay (Wantai, Beijing, China)	>4.15 log IU/mL and LSM ≥ 9.85 kPa	31 4.38 ± 0.56	75 4.03 ± 0.56	therapy-induced	31 ETV, 25LdT, 50 TNF 96 weeks	96 weeks
Lin, 2020 [28], China NR	40	PEG-IFNα-2b + ETV group 28.75 ± 4.83 PEG-IFNα-2b + TNF group 29.94 ± 5.16	PEG-IFNα-2b + ETV group 15/5 PEG-IFNα-2b + TNF group 17/3	Asia (China)	No No No	NR	total anti-HBc NR	CMIA (Abbott, Chicago, IL, USA)	NR	2 (PEG-IFNα-2b + ETV group) 8 (PEG-IFNα-2b + TNF group) NR	30 NR	therapy-induced	PEG IFN alfa 2b for 12 weeks, followed by 48 weeks of PEG IFN alfa 2b + ETV or PEG IFN alfa 2b + TNF 12 weeks	48 weeks
Fang, 2021 [29], China NR	140	overall 26.75 ± 6.99 seroclearance 25.33 ± 6.72 without seroclearance 27.32 ± 7.04	overall 103/37 seroclearance 27/13 without seroclearance 76/24	Asia (China)	No No NR	overall 51B, 89C seroclearance 16B, 24C without seroclearance 35B, 65C	total anti-HBc log IU/mL	double-sandwich immunoassay (Wantai, Beijing, China)	≥4.47 log IU/mL (30,000 IU/mL) and HBeAg level < 800 S/Co and ALT ratio × ULN ≥ 4—higher % of HBeAg seroclearance	40 4.58 ± 0.50	100 4.13 ± 0.69	therapy-induced	PEG IFN 48 weeks	72 weeks
Li, 2021 [30], China NR	93	overall 36.2 ± 9.2 seroclearance 36.0 ± 8.3 without seroclearance 36.2 ± 9.6	overall 74/19	Asia (China)	NR No No	NR	total anti-HBc log IU/mL	double-sandwich immunoassay (Wantai, Beijing, China)	NR	29 3.7 (3.2, 3.9)	64 3.4 (3.1, 3.8)	therapy-induced	ETV 78 weeks	78 weeks
Shang, 2021 [31], China Retrospective cohort	260	overall 27.88 ± 5.93 seroclearance 25.89 ± 5.43 without seroclearance 28.29 ± 5.96	overall 177/83 seroclearance 27/17 without seroclearance 150/66	Asia (China)	No No No	NR	total anti-HBc S/Co	CMIA	NR	44 11.97 (8.54–13.29)	216 11.77 (10.20–13.37)	therapy-induced	PEG IFN >4 weeks	until EOT
Zhang, 2021 [32], China NR	21	32.41 ± 9.46	19/8	Asia (China)	No No No	10B, 17C	total anti-HBc log IU/mL	double-sandwich immunoassay (Wantai, Beijing, China)	baseline ≥ 3.1 log IU/mL for HBeAg seroclearance after 10 years	19 3.17 ± 0.56	8 2.24 ± 0.86	therapy-induced	ETV 10 years	10 years
Zhao, 2021 [33], China Retrospective cohort	217	overall 35.5 (30.0–41.0) seroclearance 35.1 (30.5–38.5) without seroclearance 35.6 (39.0–42.0)	overall 160/57 seroclearance 14/8 without seroclearance 146/49	Asia (China)	NR No No	NR	total anti-HBc S/Co	commercial kit (Abbott, Wiesbaden, Germany)	baseline qAnti-HBc > 11.1 S/Co, HBeAg ≤ 3.1 log S/Co, and ALT > 152.8 U/L had highest % of HBeAg seroclearance	22 11.8 (10.8–12.5)	195 9.6 (8.3–10.8)	therapy-induced	164 ETV (HBeAg seroclearance/without HBeAg seroclearance 14/150), 53 TNF (HBeAg seroclearance/without HBeAg seroclearance 8/45) 48 weeks	48 weeks
Brakenhoff, 2022 [34], Netherlands Randomized controlled clinical trial	286 total 91 add-on PEG-IFN group 195 de novo PEG-INF group	add-on PEG-IFN group 30 (24–38) de novo PEG-INF group 33 (25–44)	add-on PEG-IFN group 65/26 de novo PEG-INF group 153/42	add-on PEG-IFN group: Caucasian (*n* = 33) Asian (*n* = 56) other (*n* = 2) de novo PEG-INF group: Caucasian (*n* = 149) Asian (*n* = 31) other (*n* = 15)	NR NR NR	add-on PEG-IFN group: 4A, 21B, 35C, 31D de novo PEG-INF group: 74A, 15B, 23C, 76D, 7 other	total anti-HBc log IU/mL	two-step sandwich CLEIA (Fujirebio, Tokyo, Japan)	baseline > 4.00 log IU/mL—higher probability of HBeAg seroclearance	16 add-on PEG-IFN group; 78 de novo PEG-INF group both groups 3.92 ± 0.27 log IU/mL	90 add-on PEG-IFN group; 117 de novo PEG-INF group both groups 3.72 ± 0.53 log IU/mL	therapy-induced	add-on PEG-IFN group: ETV 24 weeks than ETV + PEG IFN alfa 2a to 48 week; de novo PEG-INF group: PEG IFN + LAM 52 weeks	add-on PEG-IFN group: 48 weeks, de novo PEG-INF group: 76 weeks

* Age was presented as it was reported in the original article (mean ± sd, mean ± se, med (min–max), med (Q1–Q3), med (IQR)), ** value of qAnti-HBc was presented as it was reported in the original article (mean ± sd, med (min–max), med (IQR)). Abbreviations: NR—not reported; qAnti-HBc—quantitation of HBV core antibodies; PEG IFN—pegylated interferon; NA or NUC—nucleos(t)ide analogues; LAM—lamivudine; LdT—telbivudine; ADF—adefovir; ETV—entecavir; TNF—tenofovir; ALT—alanine aminotransferase; ULN—upper limit of normal; HBcrAg—hepatitis B core-related antigen; IU—international unit; PEIU—Paul Erlich Institute unit; S/Co—sample/cut-off; LSM—liver stiffness measurement; CMIA—chemiluminescent microparticle immunoassay; CLEIA—chemiluminescent enzyme immunoassay.

**Table 2:** Quality assessment.

Study	Selection Domain	Comparability Domain	Outcome/Exposure Domain	Quality
Hou, 2015 [17]	☆☆☆☆	☆	☆☆☆	Good
Wang, 2015 [18]	☆☆☆	☆	☆☆☆	Good
Gao, 2016 [19]	☆☆☆	☆	☆☆☆	Good
Zhu, 2016 [20]	☆☆☆	☆	☆☆☆	Good
Xu, 2017 [21]	☆☆☆☆	☆	☆☆☆	Good
Cai, 2018 [22]	☆☆☆	☆	☆☆☆	Good
Liao, 2018 [23]	☆☆☆		☆☆☆	Fair
Chen, 2019 [24]	☆☆☆☆	☆	☆☆☆	Good
Shen, 2019 [25]	☆☆☆	☆	☆☆☆	Good
Dezanet, 2020 [26]	☆☆☆		☆☆☆	Fair
Fu, 2020 [27]	☆☆☆	☆	☆☆☆	Good
Fang, 2021 [29]	☆☆☆	☆	☆☆☆	Good
Li, 2021 [30]	☆☆☆☆	☆	☆☆☆	Good
Shang, 2021 [31]	☆☆☆	☆	☆☆☆	Good
Zhang, 2021 [32]	☆☆☆	☆	☆☆☆	Good
Zhao, 2021 [33]	☆☆☆	☆	☆☆☆	Good
Brakenhoff, 2022 [34]	☆☆☆☆	☆	☆☆☆	Good

## Data Availability

The data presented in this study are available on request from the corresponding author.

## Supplementary Materials

The following supporting information can be downloaded at: https://www.mdpi.com/article/10.3390/v16071121/s1, Figure S1. Funnel plot for the outcome–the difference in the level of qAnti-HBc antibodies between HBV patients with and without HBeAg seroclearance; Figure S2. Funnel plot for the outcome–the difference in the level of qAnti-HBc antibodies between HBV patients originated from Asia with and without HBeAg seroclearance; Figure S3. Funnel plot for the outcome–the difference in the level of qAnti-HBc antibodies between adult HBV patients with and without therapy-induced HBeAg seroclearance; Figure S4. Funnel plot for the outcome–the difference in the level of qAnti-HBc antibodies between adult HBV patients originated from Asia with and without therapy-induced HBeAg seroclearance; Figure S5. Funnel plot for the outcome–the difference in the level of qAnti-HBc antibodies between HBV patients with and without HBeAg seroclearance (sensitivity analysis, without S/Co studies); Figure S6. Funnel plot for the outcome–the difference in the level of qAnti-HBc antibodies between HBV patients originated from Asia with and without HBeAg seroclearance (sensitivity analysis, without S/Co studies); Figure S7. Funnel plot for the outcome–the difference in the level of qAnti-HBc antibodies between adult HBV patients with and without therapy-induced HBeAg seroclearance (sensitivity analysis, without S/Co studies); Figure S8. Funnel plot for the outcome–the difference in the level of qAnti-HBc antibodies between adult HBV patients originated from Asia with and without therapy-induced HBeAg seroclearance (sensitivity analysis, without S/Co studies); Figure S9. Sensitivity analysis for the meta-analysis of the differences in level of qAnti-HBc antibodies between HBV patients with and without HBeAg seroclearance; Figure S10. Sensitivity analysis for the meta-analysis of the differences in level of qAnti-HBc antibodies between HBV patients originated from Asia with and without HBeAg seroclearance; Figure S11. Sensitivity analysis for the meta-analysis of the differences in level of qAnti-HBc antibodies between adult HBV patients with and without therapy-induced HBeAg seroclearance; Figure S12. Sensitivity analysis for the meta-analysis of the differences in level of qAnti-HBc antibodies between adult HBV patients originated from Asia with and without therapy-induced HBeAg seroclearance; Table S1. Additional patient characteristics. References [16,17,18,19,20,21,22,23,24,25,26,27,28,29,30,31,32,33,34] were cited in the supplementary materials.
