# Supplementary material for: Quantitative HBV Core Antibodies as a Prognostic Marker for HBeAg Seroclearance: A Systematic Review with Meta-Analysis
Source: Viruses. 2024 Jul 12;16(7):1121. doi: 10.3390/v16071121 (PMC11281513; doi:10.3390/v16071121)
Supplement: Supplementary file 1 [file viruses-16-01121-s001.zip › viruses-3036779-supplementary.pdf]

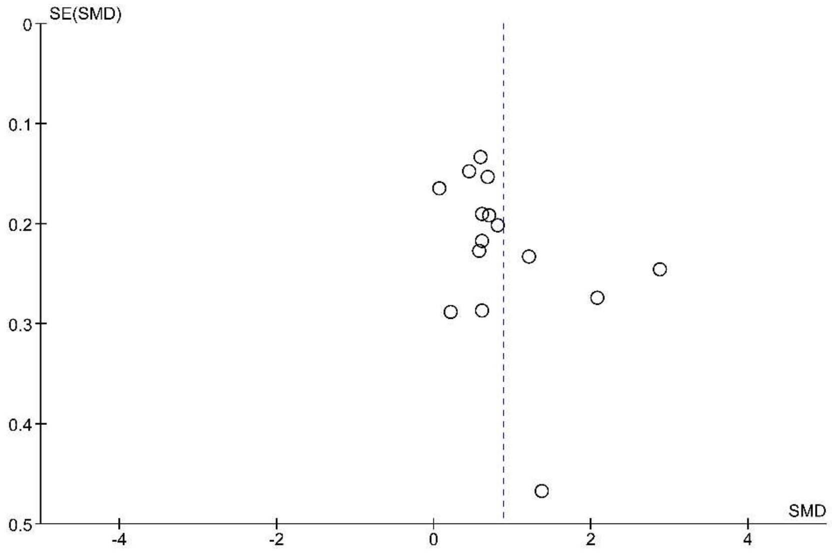

Figure S1. Funnel plot for the outcome – the difference in the level of qAnti-HBc antibodies between HBV patients with and without HBeAg seroclearance.

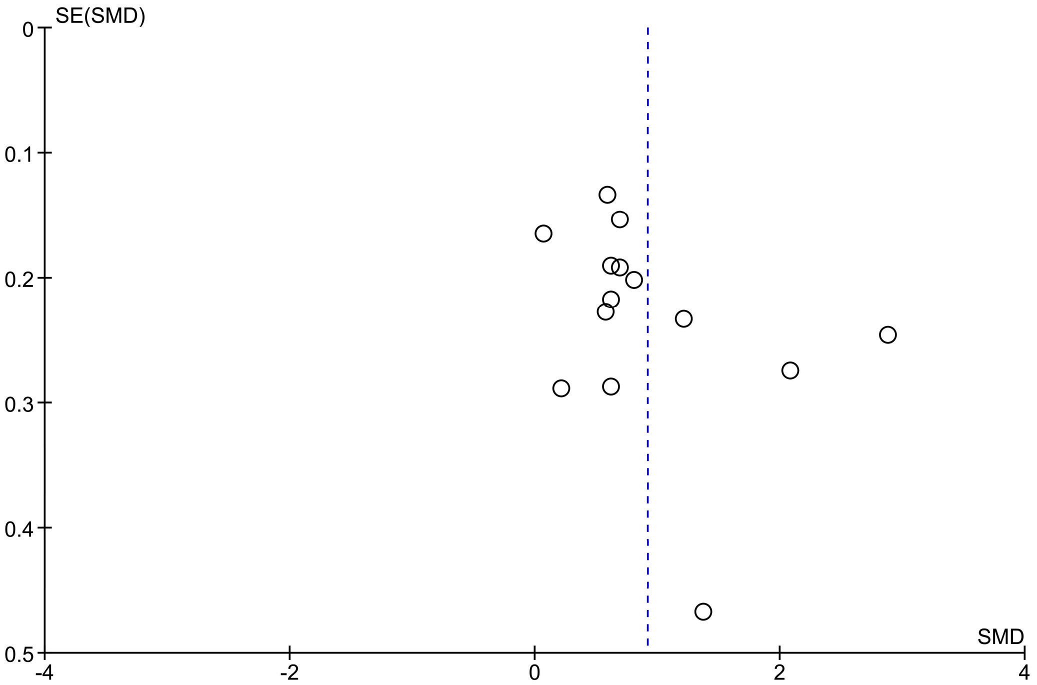

Figure S2. Funnel plot for the outcome – the difference in the level of qAnti-HBc antibodies between HBV patients originated from Asia with and without HBeAg seroclearance.

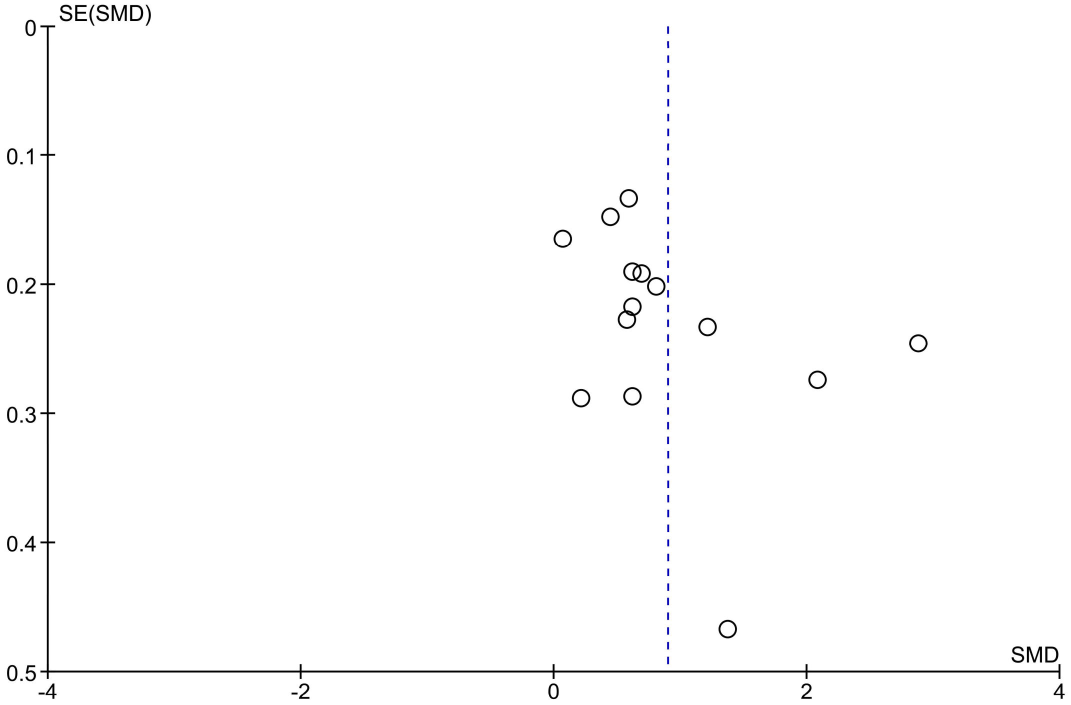

Figure S3. Funnel plot for the outcome – the difference in the level of qAnti-HBc antibodies between adult HBV patients with and without therapy-induced HBeAg seroclearance.

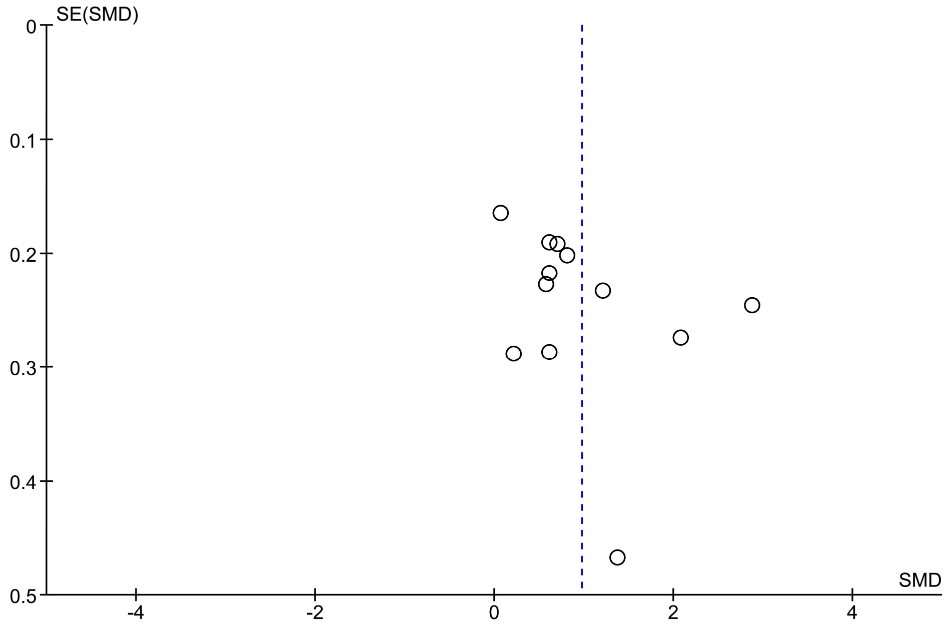

Figure S4. Funnel plot for the outcome – the difference in the level of qAnti-HBc antibodies between adult HBV patients originated from Asia with and without therapy-induced HBeAg seroclearance.

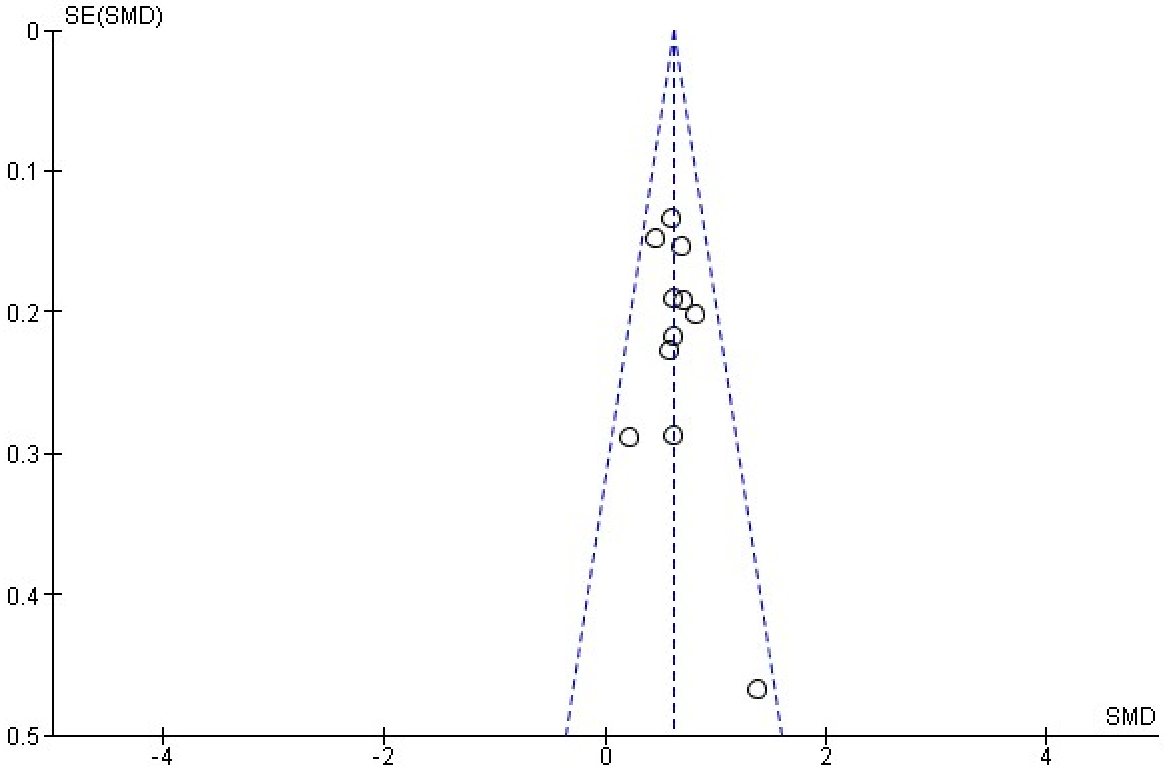

Figure S5. Funnel plot for the outcome – the difference in the level of qAnti-HBc antibodies between HBV patients with and without HBeAg seroclearance (sensitivity analysis, without S/Co studies).

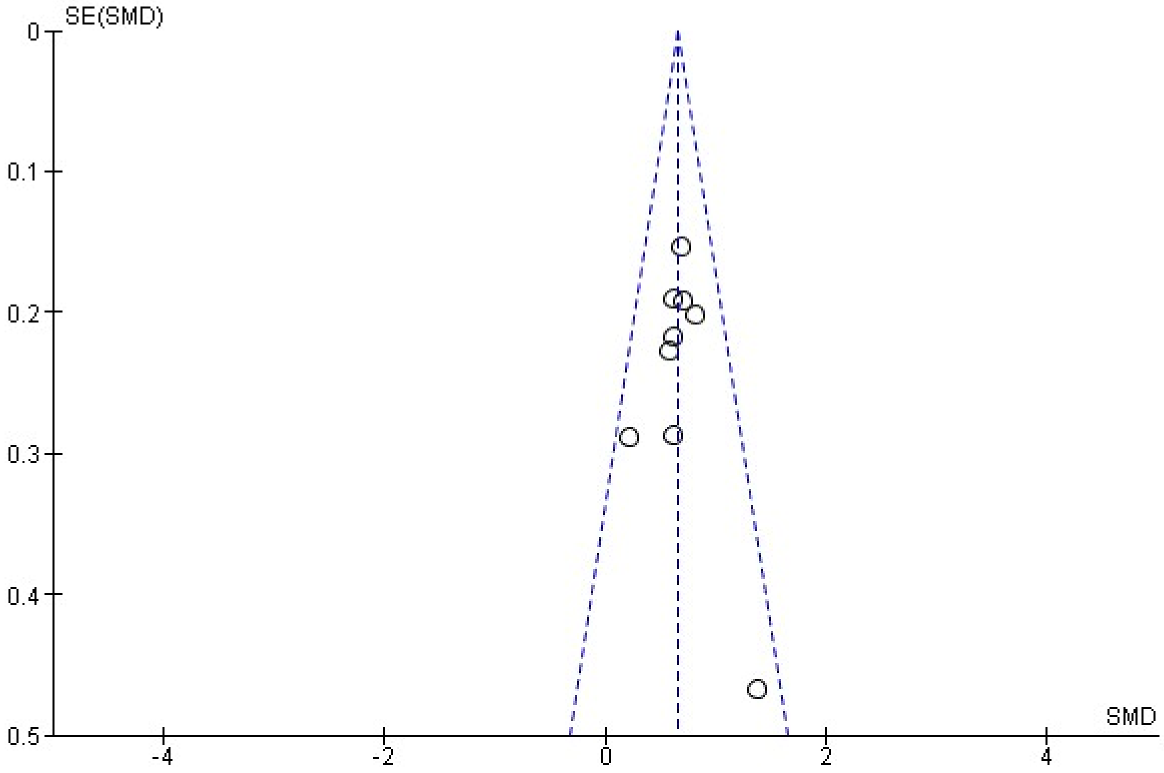

Figure S6. Funnel plot for the outcome – the difference in the level of qAnti-HBc antibodies between HBV patients originated from Asia with and without HBeAg seroclearance (sensitivity analysis, without S/Co studies).

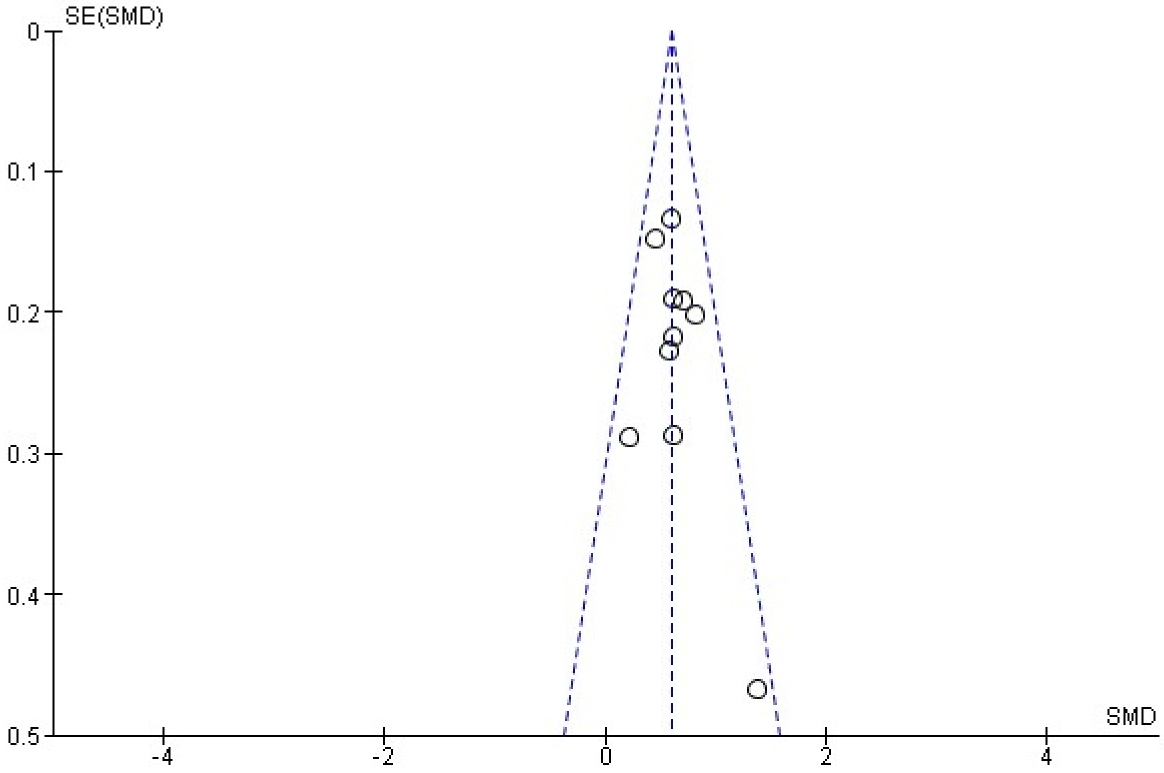

Figure S7. Funnel plot for the outcome – the difference in the level of qAnti-HBc antibodies between adult HBV patients with and without therapy-induced HBeAg seroclearance (sensitivity analysis, without S/Co studies).

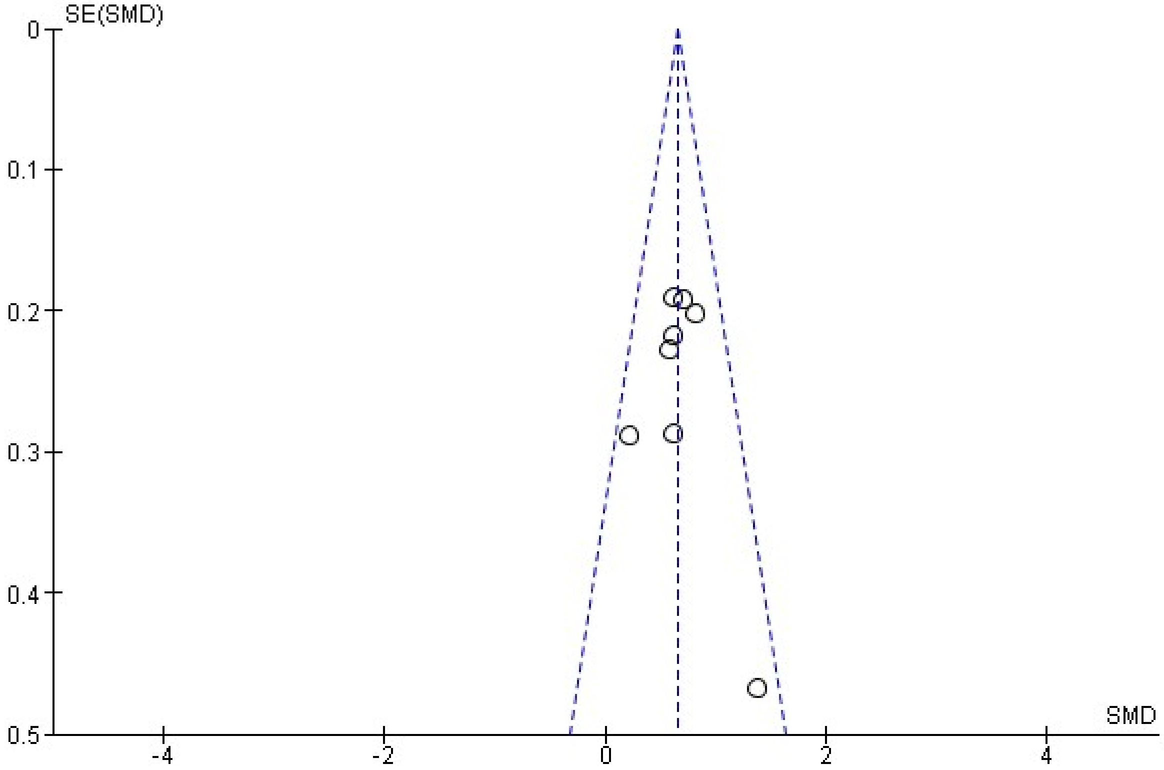

Figure S8. Funnel plot for the outcome – the difference in the level of qAnti-HBc antibodies between adult HBV patients originated from Asia with and without therapy-induced HBeAg seroclearance (sensitivity analysis, without S/Co studies).

Table S1. Additional patient characteristics.

**Study characteristics**	**Baseline values**							**qAnti-HBc**		**HBeAg seroclearance**		**HBsAg seroclearance**	
First author, year<br>Study design	AST<br><br>ALT	HBV DNA	HBsAg level	HBV RNA	HBcrAg	HBeAg level	Baseline overall qAnti-HBc values	qAnti-HBc value change from baseline	Correlation of qAnti-HBc with other parameters	Method for HBeAg detection	Parameters associated with HBeAg seroclearance	Number of HBV patients with HBsAg seroclearance	Method for HBsAg quantitation
Fan, 2015 [16]<br>Retrospective cohort	NR<br><br>PEG IFN cohort 200.0±172.7 U/mL<br>NUC cohort 192.4±172.7 U/mL	PEG IFN cohort 8.4±1.3 log copies/mL<br>NUC cohort 8.5±1.1 log copies/mL	PEG IFN cohort 4.0±0.7 log IU/mL<br>NUC cohort 4.2±0.7 log IU/mL	NR	NR	PEG IFN cohort 2.4±1.0 log PEIU/mL<br>NUC cohort 2.6±0.9 log PEIU/mL	PEG IFN cohort 4.3±0.5 log IU/mL<br>NUC cohort 4.2±0.5 log IU/mL	in PEG IFN cohort: decrease from baseline in week 12 and week 24 ; In NUC cohort: decrease from baseline to week 104	NR	PEG IFN cohort: ECL (Roche Diagnostics, Burgess Hill, UK)<br>NUC cohort: CMIA (Abbott,Chicago, IL, US)	qAnti-HBc level, HBV DNA, ALT (PEG IFN cohort), qAnti-HBc level, HBV DNA, age (NUC cohort)	NR	NR
Hou, 2015 [17]<br>Multicenter, randomized, double-blind, controlled phase II clinical trial	overall 104.88±84.20 U/L<br>seroclearance 142.21±126.31 U/L<br>without seroclearance 89.94±53.67 U/L<br><br>overall 185.72±142.90 U/L<br>seroclearance 242.66±201.97 U/L<br>without seroclearance	overall 7.68±0.76 log IU/mL<br>seroclearance 7.55±0.57 log IU/mL<br>without seroclearance 7.74±0.82 log IU/mL	overall 4.08±0.54 log IU/mL seroclearance 3.93±0.50 log IU/mL<br>without seroclearance 4.14±0.55 log IU/mL	NR	NR	overall 2.77±0.55 log S/Co, seroclearance 2.45±0.74 log S/Co without seroclearance 2.90±0.39 log S/Co	4.29±0.55 log IU/mL	qAnti-HBc continuously declined during therapy and rebound during follow-up	correlation with baseline, on-treatment and follow-up ALT level	CMIA (Abbott,Chicago, IL, US)	qAnti-HBc level and qHBeAg level	NR	NR

	162.95±103.77 U/L												
Wang, 2015 [18] Prospective cohort	NR<br><br>overall 214.25 ± 17.38 U/L seroclearance 226.23 ± 19.19 U/L without seroclearance 208.62 ± 23.94 U/L	overall 7.16 ± 0.07 log copies/mL seroclearance 6.93 ± 0.14 log copies/mL without seroclearance 7.27 ± 0.09 log copies/mL	overall 4.11 ± 0.07 log IU/mL seroclearance 3.93 ± 0.14 log IU/mL without seroclearance 4.20 ± 0.07 log IU/mL	NR	NR	overall 2.55 ± 0.07 log S/Co seroclearance 2.30 ± 0.13 log S/Co without seroclearance 2.67 ± 0.07 log S/Co	11.13 ± 0.15 S/Co	qAnti-HBc declined during therapy but decline values were not significantly different between patients who had undergone HBeAg seroclearance and those who had not	NR	commercially available kit (Abbott, Chicago, IL, US)	qAnti-HBc >11.4 S/Co , ALT level > 4 × ULN and HBeAg ≤ 500 S/CO - independent predictors	NR	NR
Gao, 2016 [19] Retrospective cohort	NR<br><br>overall 213.73 ± 157.17 U/L seroclearance 216.49 ± 153.18 U/L without seroclearance 213.05 ± 159.38 U/L	overall 8.16 ± 1.34 log copies/mL, seroclearance 8.55 ± 0.91 log copies/mL without seroclearance 8.07 ± 1.41 log copies/mL	overall 3.95 ± 0.83 log IU/mL seroclearance 3.93 ± 0.60 log IU/mL without seroclearance 3.96 ± 0.88 log IU/mL	NR	NR	overall 2.24 ± 1.31 log PEIU/mL seroclearance 2.36 ± 1.41 log PEIU/mL without seroclearance 2.21 ± 1.29 log PEIU/mL	4.77 ± 0.46 log IU/mL	decline from baseline to week 96; anti-HBc, 4.77 ± 0.46 lg IU/mL to 3.45 ± 0.65 lg IU/mL	NR	CMIA (Abbott,Chicago, IL, US)	HBeAg and HBeAg decline	1	CMIA (Abbott,Chicago, IL, US)
Xu, 2017 [20] Retrospective cohort	NR<br><br>overall 152.96±95.50 U/L seroclearance 155.35±103.6 U/L without seroclearance 152.16±93.13 U/L	overall 7.69±0.96 log IU/mL seroclearance 7.60±0.93 log IU/mL without seroclearance 7.72±0.98 log IU/mL	overall 4.41±0.70 log IU/mL seroclearance 4.19±0.87log IU/mL without seroclearance 4.48±0.61 log IU/mL	NR	NR	NR	4.78±0.77 log IU/mL	qAnti-HBc declined continuously during therapy, patients with baseline level >4.65 log IU/mL had higher values continuously	NR	commercially available kit (Abbott, Chicago, IL, US)	qAnti-HBc, gender and age - the independent predictors at week 144, qAnti-HBc and gender- independent	NR	NR

											predictors at week 240		
Zhu, 2016 [21] Prospective cohort	NR<br><br>overall 4.28 (3.17–7.32)xULN (40 U/L) seroclearance 4.84 (3.24–8.13)xULN (40 U/L) without seroclearance 4.03 (2.79–5.34)xULN (40 U/L)	overall 7.12 ± 0.19 log copies/mL seroclearance 6.90 ± 0.34 log copies/mL without seroclearance 7.27 ± 0.22 log copies/mL	overall 4.15 ± 0.15 log IU/mL seroclearance 4.01 ± 0.28 log IU/mL without seroclearance 4.26 ± 0.17 log IU/mL	NR	NR	overall 2.91 (2.49–3.09) log S/Co seroclearance 2.68 (1.77–3.07) log S/Co without seroclearance 3.00 (2.79–3.12) log S/Co	11.07 ± 0.44 S/Co	qAnti-HBc declined continuously during therapy and follow-up and was significantly higher in seroclearance group than in group without seroclearance at baseline and week 52	NR	EIA (Abbott, Chicago, IL, US)	baseline HBeAg ≤ 500 S/CO, anti-HBc > 10.7 S/CO, and ALT > 5 × ULN	7	EIA (Abbott, Chicago, IL, US)
Cai, 2018 [22] Multicenter, randomized, controlled clinical trial	NR<br><br>LAM+ADF group 226.1 ± 126.9 U/mL ETV group 276.1 ± 144.4 U/mL	LAM+ADF group 7.99 ± 0.69 log IU/mL ETV group 8.03 ± 0.58 log IU/mL	NR	NR	NR	NR	LAM+ADF group 4.06 ± 0.57 log IU/mL ETV group 4.13 ± 0.61 log IU/mL	decline from baseline in seroclearance group from 3.69 ± 0.39 log IU/mL at week 24 to 3.42 ± 0.46 log IU/mL at week 48	baseline qAnti-HBc positively associated with ALT levels, 4.25 ± 0.61 log IU/mL in those with ALT 5x ULN vs. 3.94 ± 0.47 log IU/mL in those with lower ALT	CMIA (Abbott,Chicago, IL, US)	baseline serum qAnti-HBc level - the only independent strong predictor for HBeAg seroclearance at week 96	NR	NR

Liao, 2018 [23] NR	NR<br>NR	all patients <40 IU/mL	seroclearance 2.95 (2.74, 3.43) log IU/mL without seroclearance 3.40 (3.27, 4.19) log IU/mL	seroclearance 4.39 (3.61, 5.32) log IU/mL without seroclearance 4.04 (3.58, 4.69) log IU/mL	seroclearance 5.52 (4.95, 6.31) log U/mL without seroclearance 5.75 (5.64, 6.22) log U/mL	seroclearance 3.16 (1.47, 71.18) COI without seroclearance 6.96 (4.23, 9.61) COI	2.66 (2.00, 3.01) log IU/mL	difference between start and end point: 0.22 (0.00, 1.06) (seroclearance group), 0.00 (0.00, 0.00)(group without seroclearance)	weak correlation with HBsAg level, HBcrAg , HBV RNA	ECL (Roche Diagnostics, Burgess Hill, UK)	age, HBsAg level, value of HBcrAg decline and HBV RNA	1	ECL (Roche Diagnostics, Burgess Hill, UK)
Chen, 2019 [24] NR	NR<br>overall 10 (7-15) U/mL seroclearance 11 (8-16) U/mL without seroclearance 9 (7-14) U/mL	overall 7.66 (7.19-8.18) log IU/mL seroclearance 7.88 (7.14-8.27) log IU/mL without seroclearance 7.66 (7.23-8.12) log IU/mL	overall 4.67 (4.43-4.86) log IU/mL seroclearance 4.63 (4.27 -4.74) log IU/mL without seroclearance 4.73 (4.52 -4.92) log IU/mL	NR	NR	NR	2.56 (1.82-2.85) log IU/mL	in seroclearance group: mean anti-HBc level decreased from 2.68 log IU/mL at 5 years of age to 2.15 log IU/mL at 25 years of age ($P < 0.001$); in group without seroclearance : mean qAnti-HBc level increased from 1.58 log IU/mL at 5 years of age to 2.51 log IU/mL at 25 years of age ($P < 0.001$)	correlation with baseline ALT if ALT was >30 U/mL	EIA (Abbott, Chicago, IL, US)	baseline qAnti-HBc titer of >500 IU/ mL, genotype B and B + C, and a baseline HBsAg titer of ≤4.8 log10 IU/mL	NR	NR

Shen, 2019 [25] Retrospective cohort	NR<br><br>Training cohort 4.4 ± 6.8 ULN Validation cohort 4.5 ± 7.0 ULN	Training cohort 6.8 ± 1.6 log IU/mL Validation cohort 6.9 ± 1.5 log IU/mL	Training cohort 3.5 ± 0.9 log IU/mL Validation cohort 3.5 ± 0.9 log IU/mL	NR	NR	NR	Training cohort 4.4 ± 0.6 log IU/mL Validation cohort 4.4 ± 0.7 log IU/mL	NR	NR	NR	younger age, lower HBsAg, higher qAnti-HBc, and ALT levels	NR	NR
Dezanet, 2020 [26] Prospective cohort	44 (31–78) IU/mL<br><br>63 (39–97) IU/L	6.6 (4.2–7.6) log IU/mL	4.7 (4.3–5.1) log IU/mL	NR	7.8 (7.0–8.2) log U/mL	862 (328–1099) PEIU/mL	2.8 (1.1–4.0) log PEIU/mL	decline −0.017 log PEIU/mL/month for those with HBeAg seroclearance, −0.010 log PEIU/mL/month for those without HBeAg seroclearance	higher baseline qAnti-HBc correlated with older age, no AIDS-defining illness and higher CD4+ count	ECL (Roche Diagnostics, Burgess Hill, UK) and CMIA (Abbott,Chicago, IL, US)	higher baseline qAnti-HBc and lower HBcrAg level	3	CMIA (Abbott,Chicago, IL, US)
Fu, 2020 [27] Prospective cohort	overall 54.7 ± 13.7 U/mL seroclearance 53.2 ± 16.0 U/mL without seroclearance 55.3 ± 12.6 U/mL<br><br>overall 65.7 ± 12.4 U/mL seroclearance 65.0 ± 13.3 U/mL without	seroclearance 5.92 ± 1.04 log IU/mL without seroclearance 5.37 ± 0.92 log IU/mL	overall 3.69 ± 0.54 log IU/mL seroclearance 3.63 ± 0.56 log IU/mL without seroclearance 3.71 ± 0.54 log IU/mL	NR	NR	NR	NR	significant decline of qAnti-HBc in seroclearance and group without seroclearance	slight correlation between baseline qAnti-HBc and LSM	CMIA (Abbott,Chicago, IL, US)	baseline qAnti-HBc and LSM	NR	NR

	seroclearance 66.0 ± 12.1 U/mL												
Lin, 2020 [28] NR	PEG IFNα-2b+ETV group 100.15±96.808 U/L PEG IFNα-2b+TNF group 77.94±52.372 U/L PEG IFNα-2b+ETV group 187.95±150.906 U/L PEG IFNα-2b+TNF group 143.69±115.446 U/L	PEG IFNα-2b+ETV group 6.9205±1.81974 log IU/mL PEG IFNα-2b+TNF group 7.426±0.99468 log IU/mL	PEG IFNα-2b+ETV group 4.0173±0.81801 log IU/mL PEG IFNα-2b+TNF group 4.2604±0.63169 log IU/mL	NR	NR	PEG IFNα-2b+ETV group 2.607±0.7371 log S/Co PEG IFNα-2b+TNF group 2.809±0.81298 log S/Co	NR	NR	NR	CMIA (Abbott,Chicago, IL, US)	NR	NR	NR
Fang, 2021 [29] NR	NR overall 4.66±3.56 ratio x ULN (40 U/L) seroclearance 6.13±5.02 ratio x ULN (40 U/L) without seroclearance 4.07±2.61 ratio x ULN (40 U/L)	overall 7.64±0.85 log IU/mL seroclearance 7.50±0.64 log IU/mL without seroclearance 7.70 ±0.92 log IU/mL	overall 4.07±0.60 log IU/mL seroclearance 3.95±0.50 log IU/mL without seroclearance 4.12±0.62 log IU/mL	NR	NR	overall 2.76±0.55 log S/Co seroclearance 2.45±0.75 log S/Co without seroclearance 2.88±0.38 log S/Co	4.26 ±0.67 log IU/mL	NR	NR	CMIA (Abbott,Chicago, IL, US)	qAnti-HBc ≥4.47 log IU/mL (30000 IU/mL), HBeAg level <800 S/Co and ALT ratio x ULN ≥4	NR	NR

Li, 2021 [30] NR	overall 1.3 (0.9, 2.5) ratio x ULN (40 U/L) seroclearance 2.0 (0.9, 3.7) ratio x ULN (40 U/L) without seroclearance 1.2 (0.9, 1.8) ratio x ULN (40 U/L)<br><br>overall 1.7 (1.0, 3.9) ratio x ULN (40 U/L) seroclearance 4.4 (0.9, 7.2) ratio x ULN (40 U/L) without seroclearance 1.5 (1.1, 2.6) ratio x ULN (40 U/L)	overall 6.8 (5.5, 7.6) log IU/mL seroclearance 6.5 (4.3, 7.6)log IU/mL without seroclearance 6.9 (5.8, 7.7) log IU/mL	overall 3.6 (3.1, 4.0) log IU/mL , seroclearance 3.6 (3.2, 4.3) log IU/mL without seroclearance 3.5 (3.1, 4.0) log IU/mL	NR	overall 7.4 (6.4, 8.1) log U/mL seroclearance 7.2 (6.1, 8.2) log U/mL without seroclearance 7.4 (6.5, 8.0) log U/mL	NR	3.5 (3.2, 3.8) log IU/mL	decline from 3.5 (3.2, 3.8) to 2.5 (2.1, 2.8) log IU/mL at week 78	NR	NR	the decline of HBcrAg - the only independent predictor of HBeAg seroclearance	NR	NR
Shang, 2021 [31] Retrospective cohort	seroclearance 155.70 (84.50–200.50) U/L without seroclearance 128.60 (58.0–155.50) U/L<br><br>seroclearance 304.73 (132.20–425.50) U/L without seroclearance 264.51 (99.75–314.50) U/L	seroclearance 6.84 (6.14–7.42)log IU/mL without seroclearance 6.88 (6.17–7.69) log IU/mL	seroclearance 3.77 (3.48–4.31) log IU/mL without seroclearance 3.94 (3.48–4.52) log IU/mL	NR	NR	seroclearance 410.33 (25.74–753.99) S/Co, without seroclearance 845.97 (320.24–1238.05) S/Co	NR	NR	NR	CMIA	treatment time - the most important factor in the XGBoost model followed by HBV DNA(log), HBeAg, anti-HBe, anti-HBc, ALT, TG, and ALP	NR	NR
Zhang, 2021 [32] NR	81.55±11.6 IU/mL	6.29±1.21 log IU/mL	NR	5.39±1.47 log copies/mL	NR	NR	3.07±0.87 log IU/mL	mild decline from baseline to 10 years, higher values	NR	NR	baseline qAnti-HBc level - the only	1	NR

	104.73±19.82 IU/mL							in seroclearance than group without seroclearance in all time points			independent predictor for HBeAg seroclearance after 10 years		
Zhao, 2021 [33] Retrospective cohort	NR<br>overall 188.6 (61.0–204.4) U/L seroclearance 223.5 (45.1–268.1) U/L without seroclearance 185.5 (61.8–192.3) U/L	overall 6.7 (5.7–7.8) log IU/mL seroclearance 6.1 (4.4–7.4) log IU/mL without seroclearance 6.8 (6.0–7.8) log IU/mL	NR	NR	NR	overall 3.0 (1.9–3.2) log S/Co seroclearance 2.7 (0.8–3.0) log S/Co without seroclearance 3.0 (2.0–3.2) log S/Co	9.8 (8.4–11.2) S/Co	decline from baseline to week 48: 9.9 to 8.9 S/CO, P < 0.001	NR	commercial kits (Abbott Gmbh & Co. KG, Wiesbaden, Germany)	anti-HBc > 11.1 S/Co, HBeAg ≤3.1 log S/Co, and ALT >152. 8 U/L	NR	NR
Brakenhoff, 2022 [34] Randomised controlled clinical trial	NR<br>Add on PEG-IFN group 102 (63–169) U/L De Novo PEG-INF group 130 (89–186) U/L	Add on PEG-IFN group 2.74 ±1.49 log IU/mL De Novo PEG IFN group 8.37 ±0.83 log IU/mL	Add on PEG-IFN group 3.72 ±0.66 log IU/mL De Novo PEG IFN group 4.41±0.60 log IU/mL	Add on PEG IFN group 4.85 ±1.50 log copies/mL De Novo PEG IFN group: 6.79±1.11 log copies/mL	Add on PEG IFN group 8.11±0.76 log U/ml De Novo PEG IFN group 8.35±0.70 log U/mL	NR	Add on PEG IFN group 2.88 ±0.73 log IU/mL De Novo PEG IFN group 3.80±0.46 log IU/mL	decline from baseline to EOT in Add on PEG IFN group: 0.29(0.28) log IU/mL, in De Novo PEG IFN group: 0.25(0.36) log IU/mL	NR	NR	higher baseline levels of qAnti-HBc - a higher probability of favourable outcomes, including HBeAg seroclearance in both groups	Add on PEG IFN group: 3 ;De Novo PEG IFN group: 16	CMIA (Abbott, Chicago, IL, US)

Abbreviations: NR – not reported; qAnti-HBc – quantitation of HBV core antibodies; PEG IFN - pegylated interferon; NA or NUC – nucleos(t)ide analogues; LAM – lamivudine; ADF – adefovir; ETV – entecavir; TNF – tenofovir; AST- aspartate aminotransferase; ALT - alanine aminotransferase; ULN – upper limit of normal; HBcrAg – hepatitis B core-related antigen;; LSM – liver stiffness measurement; TG – triglyceride; ALP - alkaline phosphatase; IU- international units; PEIU – Paul-Erlich-Institute units; S/Co – sample/cut-off; COI- cut-off index; EIA –enyzme immunoassay; CMIA—chemiluminescent microparticle immunoassay; CLEIA—chemiluminescent enzyme immunoassay; ECL - electrochemical luminescence immunoassay.

Study or Subgroup	With HBeAg seroclearance Mean	SD	Total	Without HBeAg seroclearan Mean	SD	Total	Weight	Std. Mean Difference IV, Fixed, 95% CI	Year
Hou 2015	4.52	0.36	40	4.19	0.58	100	9.4%	0.62 [0.25, 1.00]	2015
Gao 2016	4.85	0.44	15	4.75	0.47	61	4.1%	0.21 [-0.35, 0.78]	2016
Xu 2017	5.22	0.46	35	4.63	0.79	104	8.4%	0.81 [0.42, 1.21]	2017
Cai 2018	4.38	0.54	16	4.02	0.58	58	4.1%	0.62 [0.06, 1.19]	2018
Liao 2018	2.71	1.21	9	2	0	7		Not estimable	2018
Chen 2019	2.76	0.55	85	2.2	0.98	97	14.6%	0.69 [0.39, 0.99]	2019
Shen 2019	4.7	0.6	85	4.3	0.7	181	19.0%	0.60 [0.33, 0.86]	2019
Fu 2020	4.38	0.56	31	4.03	0.56	75	7.2%	0.62 [0.19, 1.05]	2020
Fang 2021	4.58	0.5	40	4.13	0.69	100	9.3%	0.70 [0.32, 1.07]	2021
Zhang 2021	3.17	0.56	19	2.24	0.86	8	1.6%	1.37 [0.45, 2.29]	2021
Li 2021	3.7	0.52	29	3.4	0.52	64	6.6%	0.57 [0.13, 1.02]	2021
Brakenhoff 2022	3.92	0.27	78	3.72	0.53	117	15.6%	0.45 [0.16, 0.74]	2022
**Total (95% CI)**			**482**			**972**	**100.0%**	**0.61 [0.50, 0.73]**	

Heterogeneity: Chi² = 7.27, df = 10 (P = 0.70); I² = 0%
Test for overall effect: Z = 10.50 (P < 0.00001)

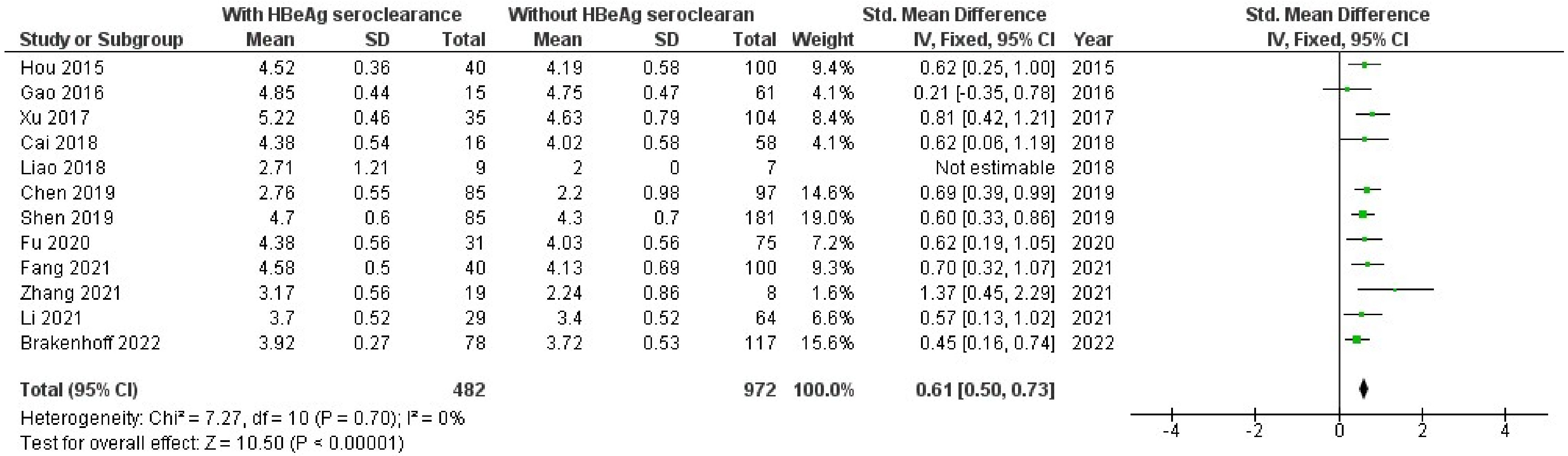

Figure S9. Sensitivity analysis for the meta-analysis of the differences in level of qAnti-HBc antibodies between HBV patients with and without HBeAg seroclearance. [17,19,21–25,27,29,30,32,34]

Study or Subgroup	With HBeAg seroclearance Mean	SD	Total	Without HBeAg seroclearance Mean	SD	Total	Weight	Std. Mean Difference IV, Fixed, 95% CI	Year
Hou 2015	4.52	0.36	40	4.19	0.58	100	14.4%	0.62 [0.25, 1.00]	2015
Gao 2016	4.85	0.44	15	4.75	0.47	61	6.3%	0.21 [-0.35, 0.78]	2016
Xu 2017	5.22	0.46	35	4.63	0.79	104	12.9%	0.81 [0.42, 1.21]	2017
Cai 2018	4.38	0.54	16	4.02	0.58	58	6.3%	0.62 [0.06, 1.19]	2018
Liao 2018	2.71	1.21	9	2	0	7		Not estimable	2018
Chen 2019	2.76	0.55	85	2.2	0.98	97	22.4%	0.69 [0.39, 0.99]	2019
Fu 2020	4.38	0.56	31	4.03	0.56	75	11.0%	0.62 [0.19, 1.05]	2020
Zhang 2021	3.17	0.56	19	2.24	0.86	8	2.4%	1.37 [0.45, 2.29]	2021
Li 2021	3.7	0.52	29	3.4	0.52	64	10.1%	0.57 [0.13, 1.02]	2021
Fang 2021	4.58	0.5	40	4.13	0.69	100	14.2%	0.70 [0.32, 1.07]	2021
**Total (95% CI)**			**319**			**674**	**100.0%**	**0.66 [0.52, 0.80]**	

Heterogeneity: Chi² = 5.59, df = 8 (P = 0.69); I² = 0%
Test for overall effect: Z = 9.12 (P < 0.00001)

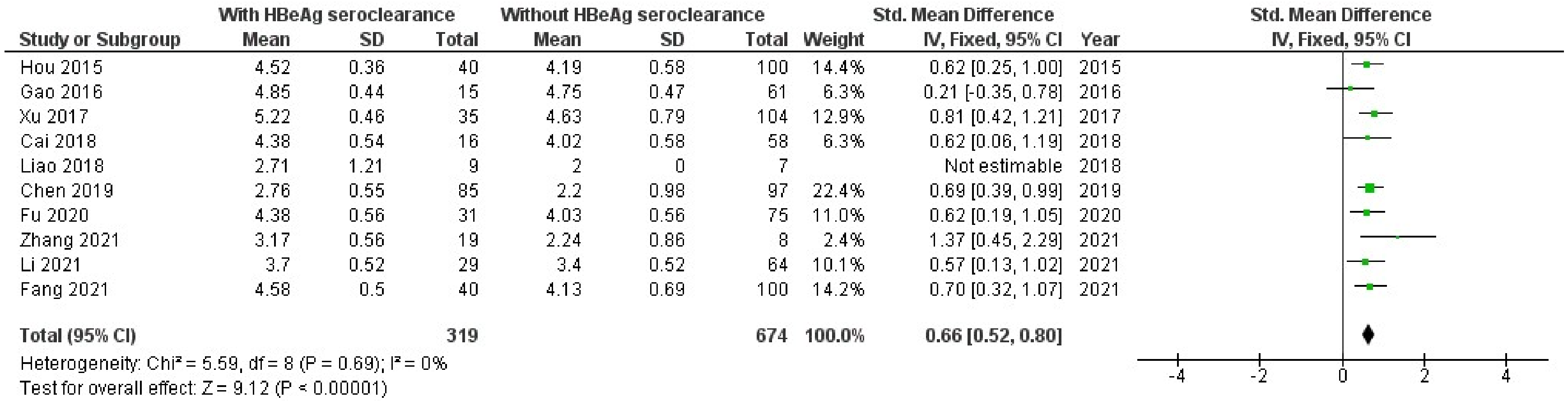

Figure S10. Sensitivity analysis for the meta-analysis of the differences in level of qAnti-HBc antibodies between HBV patients originated from Asia with and without HBeAg seroclearance [17,19,21–24,27,29,30,32].

Study or Subgroup	With HBeAg seroclearance Mean	SD	Total	Without HBeAg seroclearance Mean	SD	Total	Weight	Std. Mean Difference IV, Fixed, 95% CI	Year
Hou 2015	4.52	0.36	40	4.19	0.58	100	11.0%	0.62 [0.25, 1.00]	2015
Gao 2016	4.85	0.44	15	4.75	0.47	61	4.8%	0.21 [-0.35, 0.78]	2016
Xu 2017	5.22	0.46	35	4.63	0.79	104	9.9%	0.81 [0.42, 1.21]	2017
Liao 2018	2.71	1.21	9	2	0	7		Not estimable	2018
Cai 2018	4.38	0.54	16	4.02	0.58	58	4.9%	0.62 [0.06, 1.19]	2018
Shen 2019	4.7	0.6	85	4.3	0.7	181	22.3%	0.60 [0.33, 0.86]	2019
Fu 2020	4.38	0.56	31	4.03	0.56	75	8.4%	0.62 [0.19, 1.05]	2020
Fang 2021	4.58	0.5	40	4.13	0.69	100	10.9%	0.70 [0.32, 1.07]	2021
Li 2021	3.7	0.52	29	3.4	0.52	64	7.7%	0.57 [0.13, 1.02]	2021
Zhang 2021	3.17	0.56	19	2.24	0.86	8	1.8%	1.37 [0.45, 2.29]	2021
Brakenhoff 2022	3.92	0.27	78	3.72	0.53	117	18.3%	0.45 [0.16, 0.74]	2022
**Total (95% CI)**			**397**			**875**	**100.0%**	**0.60 [0.48, 0.73]**	

Heterogeneity: Chi² = 6.98, df = 9 (P = 0.64); I² = 0%
Test for overall effect: Z = 9.50 (P < 0.00001)

Std. Mean Difference IV, Fixed, 95% CI

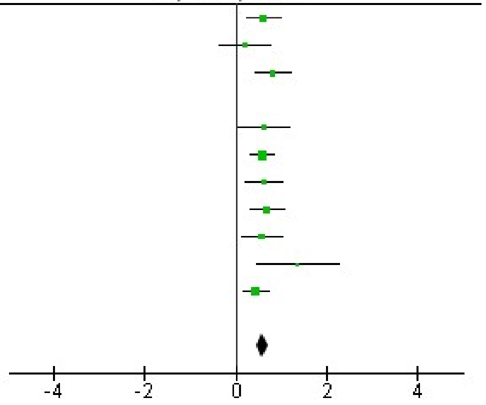

Figure S11. Sensitivity analysis for the meta-analysis of the differences in level of qAnti-HBc antibodies between adult HBV patients with and without therapy-induced HBeAg seroclearance [17,19,21-23,25,27–29,32,34].

Study or Subgroup	With HBeAg seroclearance Mean	SD	Total	Without HBeAg seroclearance Mean	SD	Total	Weight	Std. Mean Difference IV, Fixed, 95% CI	Year
Hou 2015	4.52	0.36	40	4.19	0.58	100	18.5%	0.62 [0.25, 1.00]	2015
Gao 2016	4.85	0.44	15	4.75	0.47	61	8.1%	0.21 [-0.35, 0.78]	2016
Xu 2017	5.22	0.46	35	4.63	0.79	104	16.6%	0.81 [0.42, 1.21]	2017
Liao 2018	2.71	1.21	9	2	0	7		Not estimable	2018
Cai 2018	4.38	0.54	16	4.02	0.58	58	8.2%	0.62 [0.06, 1.19]	2018
Fu 2020	4.38	0.56	31	4.03	0.56	75	14.2%	0.62 [0.19, 1.05]	2020
Li 2021	3.7	0.52	29	3.4	0.52	64	13.0%	0.57 [0.13, 1.02]	2021
Fang 2021	4.58	0.5	40	4.13	0.69	100	18.3%	0.70 [0.32, 1.07]	2021
Zhang 2021	3.17	0.56	19	2.24	0.86	8	3.1%	1.37 [0.45, 2.29]	2021
**Total (95% CI)**			**234**			**577**	**100.0%**	**0.65 [0.49, 0.81]**	

Heterogeneity: Chi² = 5.54, df = 7 (P = 0.59); I² = 0%
Test for overall effect: Z = 7.93 (P < 0.00001)

Std. Mean Difference IV, Fixed, 95% CI

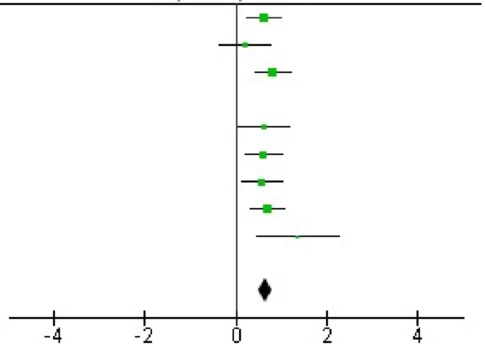

Figure S12. Sensitivity analysis for the meta-analysis of the differences in level of qAnti-HBc antibodies between adult HBV patients originated from Asia with and without therapy-induced HBeAg seroclearance [17,19,21–23,27,29,30,32].
